# Improvement of stem cell-derived exosome release efficiency by surface-modified nanoparticles

**DOI:** 10.1186/s12951-020-00739-7

**Published:** 2020-12-07

**Authors:** Dong Jun Park, Wan Su Yun, Woo Cheol Kim, Jeong-Eun Park, Su Hoon Lee, Sunmok Ha, Jin Sil Choi, Jaehong Key, Young Joon Seo

**Affiliations:** 1 Department of Otorhinolaryngology, Yonsei University Wonju College of Medicine, 20 Ilsan-ro, Wonju, Gangwon-do 26426 South Korea; 2 Research Institute of Hearing Enhancement, Yonsei University Wonju College of Medicine, Wonju, South Korea; 3 Department of Biomedical Engineering, Yonsei University, Wonju, South Korea; 4 Department of Biomedical Laboratory Science, College of Health Sciences, Yonsei University, Wonju, Republic of Korea

**Keywords:** Autophagy, Mesenchymal stem cell-derived exosome, Positively charged nanoparticle, PLGA-PEI NPs, Rab7

## Abstract

**Background:**

Mesenchymal stem cells (MSCs) are pluripotent stromal cells that release extracellular vesicles (EVs). EVs contain various growth factors and antioxidants that can positively affect the surrounding cells. Nanoscale MSC-derived EVs, such as exosomes, have been developed as bio-stable nano-type materials. However, some issues, such as low yield and difficulty in quantification, limit their use. We hypothesized that enhancing exosome production using nanoparticles would stimulate the release of intracellular molecules.

**Results:**

The aim of this study was to elucidate the molecular mechanisms of exosome generation by comparing the internalization of surface-modified, positively charged nanoparticles and exosome generation from MSCs. We determined that Rab7, a late endosome and auto-phagosome marker, was increased upon exosome expression and was associated with autophagosome formation.

**Conclusions:**

It was concluded that the nanoparticles we developed were transported to the lysosome by clathrin-mediated endocytosis. additionally, entered nanoparticles stimulated that autophagy related factors to release exosome from the MSC. MSC-derived exosomes using nanoparticles may increase exosome yield and enable the discovery of nanoparticle-induced genetic factors.
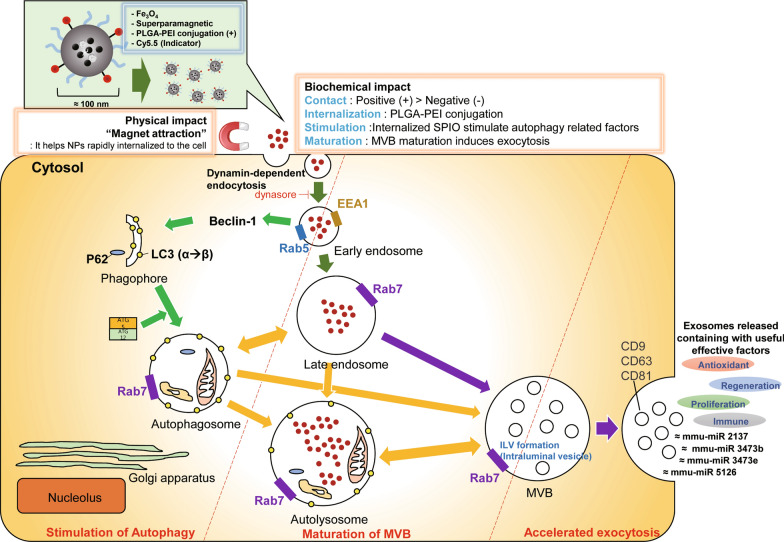

## Background

Therapeutic clinical studies using mesenchymal stem cells (MSCs) have been recently reported, and techniques using MSC-derived exosomes are being actively developed. Several studies have explored the application of cell-derived exosomes for medical purposes. For example, MSC-derived exosomes have been used to suppress immune responses or as a therapeutic treatment for cancer [1, 2]. In cell-free therapies, to increase the quantity of exosomes, biocompatible materials are now being used [3, 4]. However, the protocols for obtaining a consistent yield and an efficient exosome extraction process for medical use have not been established yet.

The yield of exosomes derived from stem cells is very low, which warrants optimization of conditions to increase yields. Sucrose gradient-based ultra-centrifugation is commonly used to obtain materials for various applications [5–7]. Extracellular vesicles (EVs) play an important role in cell-to-cell communication through the delivery of biological content, including intercellular proteins, lipids, and nucleic acids. Nanoscale EVs are difficult to maintain their morphology and function, and exosomes originating from multiple vesicle bodies (MVBs) are difficult to quantify [8, 9]. Quantification of the released exosomes is generally done by evaluating protein or microRNA (miRNA) content, although no formal method has been established [10, 11]. Exosomes are quantified by measuring the amount of total protein expressed in the exosome membrane or by confirming the expression of specific proteins, such as CD81, CD9, or CD63 [12, 13]. Techniques to extract exosomes and predict their quantity based on absorbance values, e.g. cytokine assays or enzyme-linked immunosorbent assays, have also been reported [2].

In this study, bioavailable nanoparticles (NPs) based on iron oxide with poly(lactic-co-glycolic acid), or PLGA, were used to improve the yield and expression of target molecules. We investigated the effects of NP surface charge and external stimuli on the generation of exosomes with the goal of amplifying the yield of exosomes for applied research [14, 15]. The unique antioxidant and growth factors contained in exosomes were also considered. Iron oxide-based superparamagnetic iron oxide NPs (SPIONs) are bio-stable particles that have been approved by the United States Food and Drug Administration [16, 17]. NPs encapsulated with PLGA and polyethyleneimine (PEI) are approximately 100 nm in size and are used for drug delivery and clinical applications [15, 18, 19]. Altering the surface charge of NPs has demonstrated that positively charged NPs are more readily introduced into cells compared to the negatively charged NPs [16, 20]. Furthermore, the reason for using magnetic iron oxide NPs in this study was to introduce a higher number of NPs into cells using a magnetic force. A clinical study reported the use of magnetism to deliver drugs into cells [19]. Here we utilized these iron oxide-based NPs to develop a technique to improve the production of exosomes from stem cells. We analyzed the mechanisms connecting the NPs and the generation of exosomes and discovered an indicator related to exosome release by stimulating MVB formation. These results provide information on how to improve the composition and yield of exosomes.

## Results

### Synthesis and characterization of polymeric clustered superparamagnetic (PCS) NPs

**Fig. 1:**
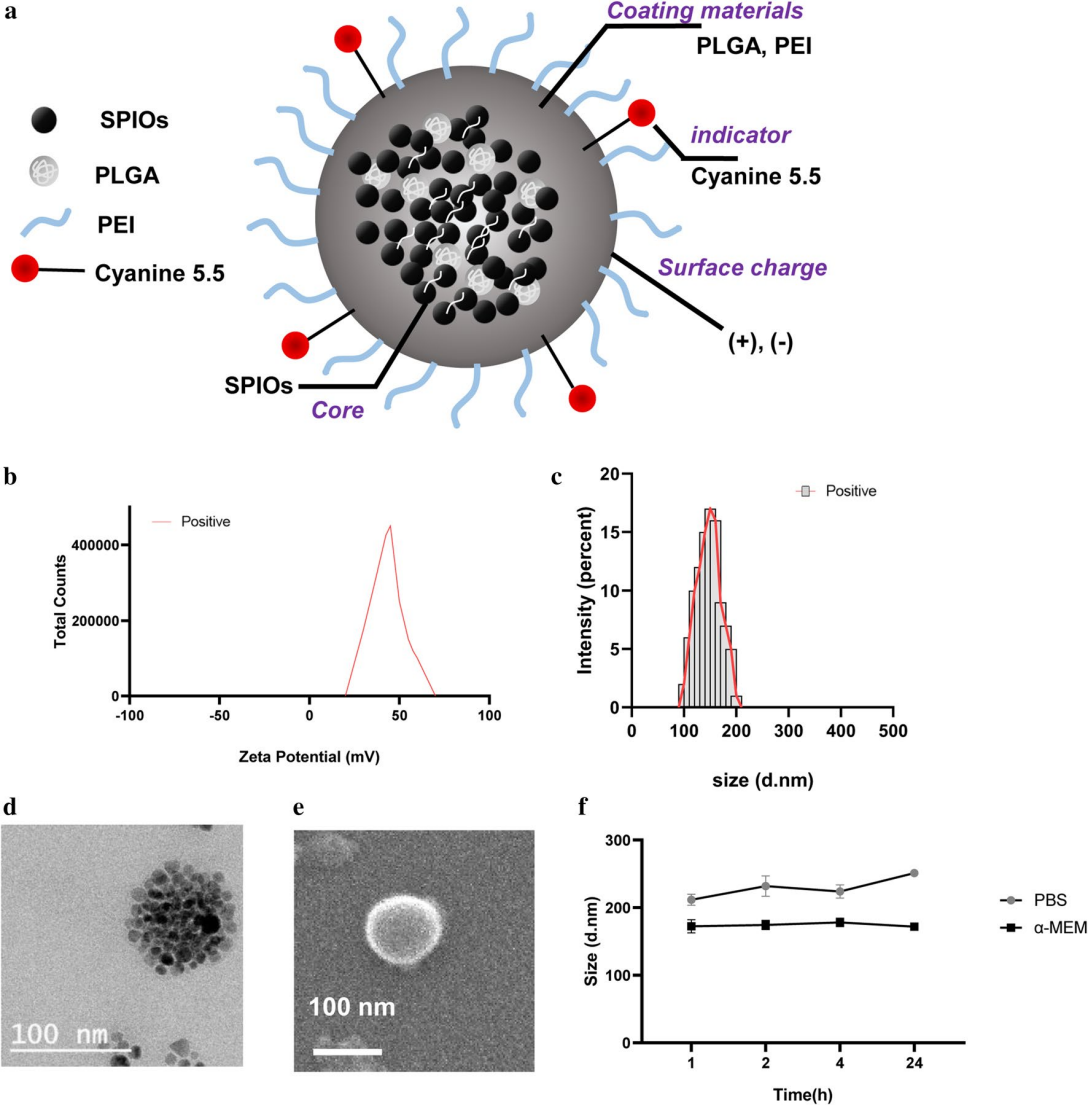
Properties of positively charged PCS NPs. **a** Schematic representation of PLGA-PEI PCS NPs. **b** ζ-potential. **c** Size. **d** TEM image. **e** SEM image. **f** Stability test of PLGA-PEI PCS NPs in the medium and PBS

Magnetic NP solution (SPIO)-based polymeric clustered superparamagnetic (PCS) NPs were prepared with different surface modifications [21]. Modifying NPs with silica or other components does not improve their physical capabilities. However, the use of SPIO has the advantage of introducing more NPs into cells [15, 22]. Surface charges vary depending on the composition of the NPs. In this study, SPIOs were composed using specific PLGA-to-PEI ratios (Additional file 1: Figure S1). This produced PLGA (negatively charged) and PLGA-PEI (PLGA 8: PEI 2; positively charged) PCS NPs. The zeta (ζ)-potential for the PLGA NPs was − 30 mV and + 35 mV for the PLGA-PEI NPs. Electron microscopy revealed that both NP types were approximately 100 nm in size (Fig. 1, Additional file 1: Figure S2). The surface properties of the NPs were also analyzed. NPs were stable in medium and phosphate-buffered saline (Fig. 1f). Detailed properties and explanations are provided in Additional file 1: Figure S2.

### Cellular uptake of PLGA-PEI PCS NPs and exosome release

**Fig. 2:**
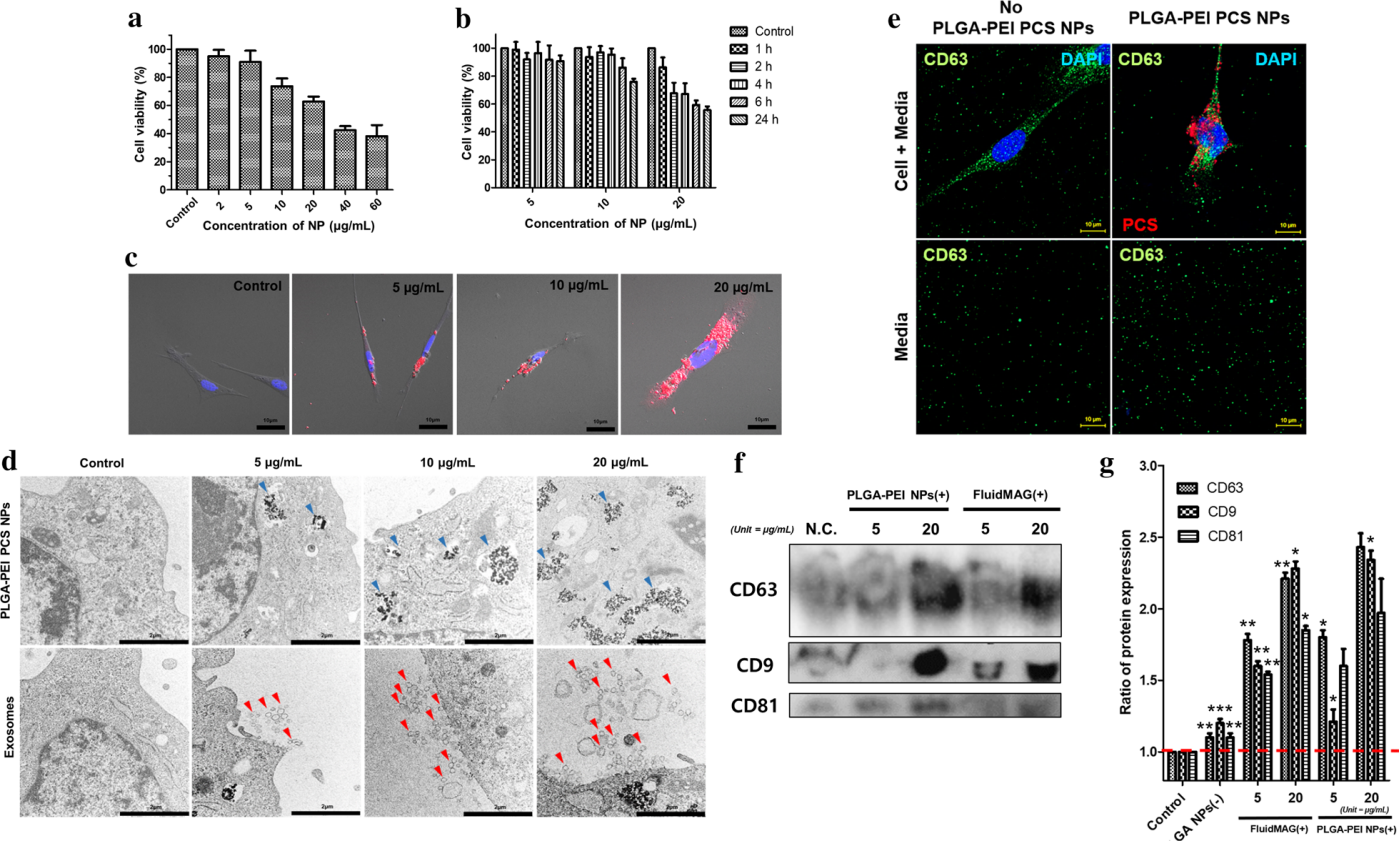
MSC viability in response to treatment with PLGA-PEI PCS NPs (+) and observation of NP internalization. Exosome expression was also observed according to internalization of PLGA-PEI PCS NPs. **a** Cell viability of MSCs treated with 2, 5, 10, 20, 40, and 60 μg/mL PCS NPs. **b** Cell viability of MSCs treated with PCS NPs during exposure for 1, 2, 4, 6, and 24 h. **c** Images of MSCs incubated with 0, 5, 10, and 20 μg/mL PCS NPs using fluorescent microscopy. **d** TEM images of PCS NPs and extracellular vesicles (exosomes and macrovesicles) in MSCs. The blue arrows depict PCS NPs and the red arrows depict extracellular vesicles. **e** The release of exosomal marker CD63 by the MSCs in media. **f** Identification of exosomal markers CD63, CD9, and CD81 released by the MSCs in media as determined by Western blot. **g** Quantification of expression levels of exosomal markers released by the MSCs. Data are mean ± SD. *p < 0.05, ***p < 0.001

To evaluate the effect of PLGA-PEI PCS NPs (+) on MSCs, we measured the viability of MSCs using the CCK-8 assay in response to different concentrations of PLGA-PEI PCS NPs (+) for 24 h. Cell viability was 90.9 ± 8.14% at 5 μg/mL concentration (Fig. 2a); however, higher concentrations of NPs decreased cell viability (73.6%). The cell viability was the highest at the 24 h time point and 5 μg/mL NP concentration (90.7 ± 4.1%) (Fig. 2b). When cells were treated with 10 μg/mL and 20 μg/mL NPs, viability decreased to 86.2% after 6 h and to 86% after 1 h, respectively. These findings indicated that the concentration of 5 μg/mL was optimal for internalization and that higher concentrations of positively charged PCS NPs were increasingly internalized by MSCs, affecting their viability (Fig. 2c).

To determine the localization of the NPs, MSCs were treated with 5, 10, and 20 μg/mL PCS NPs for 24 h and then imaged using a transmission electron microscope (TEM). The number of NPs internalized increased depending on the concentration. The NPs appeared to be internalized in clusters rather than as single particles (Fig. 2d). Furthermore, the internalization pattern of NPs was different depending on their surface charge: positively charged PCS NPs tended to aggregate for internalization, whereas negatively charged NPs tended to be singular (Additional file 1: Figure S3).

Interestingly, PLGA-PEI PCS NPs were internalized into intracellular organelles for 24 h, which was thought to greatly influence the formation of exosomes (Additional file 2). In Fig. 2d, the blue arrows depict NPs and the red arrows point to exosome and macrovesicles released from MSCs. The quantity of exosomes increased proportionally depending on the concentration of PCS NPs (Fig. 2d). The size of exosomes ranged from 91 to 147 nm (Additional file 1: Figure S4).

To define the mechanisms of exosome release, we used two types of FluidMAG NPs (positive and negative) as controls. Exosomes were detected only in cells treated with positively charged NPs (Additional file 1: Figure S3). More positively charged particles adhered to the cell surface (Additional file 1: Figure S5). Exosomes were mainly observed at concentrations of positively charged PCS NPs that induced apoptosis, with increased expression of exosome protein markers detected by immunofluorescence (Fig. 2e) and western blotting (Fig. 2f, g). Next, to examine the quality of exosomes, we evaluated exosome extraction conditions by assessing miRNA expression.

### Profiling of functional miRNAs in exosomes

**Fig. 3:**
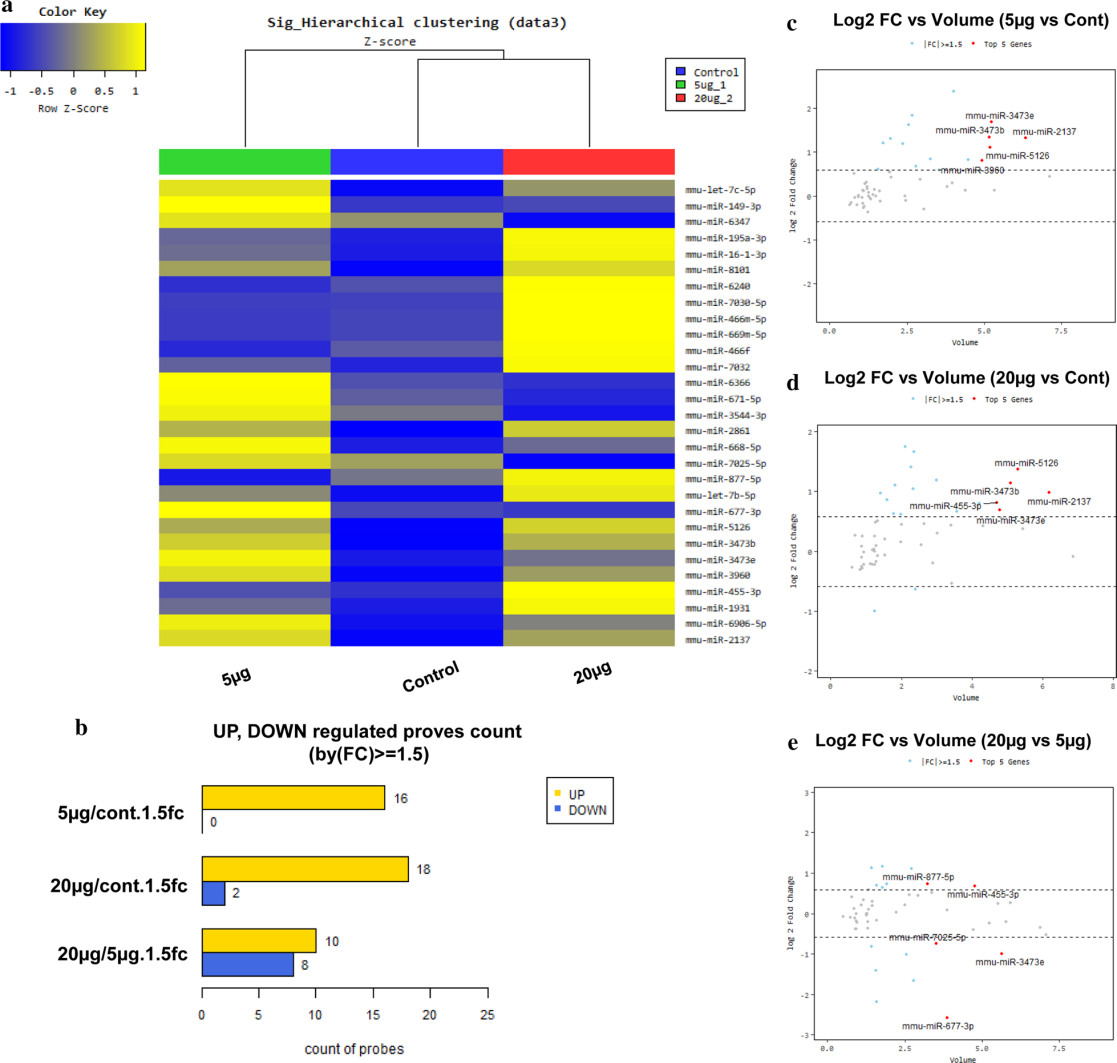
Analysis of exosomal miRNAs from MSCs exposed to 5 and 20 μg/mL PLGA-PEI PCS NPs. **a** Heat map with fold change values after different concentrations of NPs were added to MSCs for 24 h. **b** Number of miRNA probes that are increased or decreased (control and 5 μg/mL treatment, control and 20 μg/mL treatment, 5 and 20 μg/mL treatment). **c** The ratio of volume vs. fold change in the control and 5 μg/mL treatment. **d** The ratio of volume vs. fold change in the control and 20 μg/mL treatment. **e** The ratio of volume vs. fold change in the 5 and 20 μg/mL treatments

To identify the overexpressed components in the exosomes in MSCs after treatment with NPs, we analyzed the miRNA profiles. Figure 3a depicts the comparison of the levels of expressed miRNAs in exosomes as heatmaps with fold change values. Twenty-nine miRNAs with a fold change > 1.5 were selected. miRNA counts were compared between the control and 5 μg/mL, control and 20 μg/mL, and 5 and 20 μg/mL groups (Fig. 3b). We focused on miRNAs with increased expression. Comparisons based on log 2 and volume were used to select five representative miRNAs. In MSCs incubated with 5 μg/mL NPs, mmu-miR-2137, mmu-miR-3473b, mmu-miR-3473e, mmu-miR-3960, and mmu-miR-5126 displayed the highest volume and fold change values compared to the control (Fig. 3c). In MSCs incubated with 20 μg/mL NPs, mmu-miR-2137, mmu-miR-3473b, mmu-miR-3473e, mmu-miR-5126, and mmu-miR-455-3p displayed the highest volume and fold change values compared to the control (Fig. 3d). We conducted the analysis using the miRNA database and predicted the genes that each miRNA could target. In addition, the selected exosomal miRNAs were identified (Additional file 1: Figure S6) and compared with the exosomal components previously reported using the exoCarta exosome database (Table 1). The details of the miRNA analysis are summarized in Additional file 1: Figure S6. Importantly, we confirmed that the exosomes generated by the PCS NPs contained host factors affecting antioxidant properties, cell differentiation, and cell proliferation.

**Table 1:** List of predicted mRNA targets of the miRNAs

miRNA	Predicted target mRNA		Functions and characteristics	Database on ExoCerta
	Symbol	Full name		
mmu-miR-2137	*Gpx4*	Glutathione peroxidase 4	Plays a key role in protecting cells from oxidative damage by preventing membrane lipid peroxidation	o
	*Gosr1*	Golgi SNAP receptor complex member 1	Regulates intracellular reactive oxygen species levels via inhibition of p38 MAPK (MAPK11, MAPK12, MAPK13, and MAPK14)	o
	*Rab4A*	Ras-related protein Rab-4A	Protein transport. Plays a role in vesicular traffic. Mediates VEGFR2 endosomal trafficking to enhance VEGFR2 signaling	o
	*Prrx1*	Paired related homeobox 1	Enhances DNA-binding activity, and induces genes by growth and differentiation factors	Unknown
	*Pex26*	Peroxisomal biogenesis factor 26	Peroxisome biogenesis factor 1, also known as PEX1	Unknown
mmu-miR-3473b	*Rspry1*	Ring finger and SPRY domain containing 1	Extracellular region and zinc ion binding	o
	*Ephb2*	Eph receptor B2	Encodes a member of the Eph receptor family of receptor tyrosine kinase transmembrane glycoproteins	o
	*Cplx2*	Complexin 2	Refers to one of a small set of eukaryotic cytoplasmic neuronal proteins that binds to the SNARE protein complex (SNAREpin) with high affinity	o
	*Il-7*	Interleukin 7	IL-7 stimulates the differentiation of multipotent (pluripotent) hematopoietic stem cells into lymphoid progenitor cells	o
	*Rab11fip1*	RAB11 family interacting protein 1	Forms a targeting complex that recruits a group of proteins involved in membrane transport to organelles	o
mmu-miR-3473e	*St6galnac6*	ST6-*N*-acetylgalactosaminide alpha-2,6-sialyltransferase 6	Modifies ceramides on the cell surface to alter cell–cell or cell-extracellular matrix interactions	o
	*Adcy1*	Adenylate cyclase 1	Regulates various cellular functions via activating protein kinase A-dependent phosphorylation	o
	*Wnk2*	Serine/threonine-protein kinase WNK2	Plays an important role in the regulation of electrolyte homeostasis, cell signaling, survival, and proliferation	o
	*Sioa1l3*	Signal-induced proliferation-associated 1-like protein 3	Plays a critical role in epithelial cell morphogenesis, polarity, and adhesion and cytoskeletal organization in the lens	Unknown
mmu-miR-5126	*Atp6v1c2*	V-type proton ATPase subunit C 2	Subunit of the peripheral V1 complex of vacuolar ATPase	o
	*CD4*	CD4 molecules	Integral membrane glycoprotein that plays an essential role in the immune response and serves multiple functions in responses against both external and internal offenses	o
	*Pax2*	Paired box 2	Has a critical role in the development of the urogenital tract, the eyes, and the central nervous system	Unknown
	*Aire*	Autoimmune regulator	Expressed by a distinct bone marrow-derived population; induces self-tolerance through a mechanism that does not require regulatory T-cells and is resistant to innate inflammatory stimuli	Unknown
mmu-miR-455-3p	*Tti1*	TELO2-interacting protein 1 homolog	Phosphorylated at Ser-823 by CK2 following growth factor deprivation, leading to its subsequent ubiquitination by the SCF (FBXO9) complex	o
	*Hk3*	Hexokinase 3	HK3 functions to protect the cell against apoptosis. Overexpression of HK3 has resulted in increased ATP levels, decreased reactive oxygen species production, attenuated reduction in the mitochondrial membrane potential, and enhanced mitochondrial biogenesis	o
	*Sar1a*	Secretion associated Ras-related GTPase 1A	Involved in membrane trafficking. It is a monomeric small GTPase found in COPI vesicles. It regulates the assembly and disassembly of COPII coats	o
	*Fgf4*	Fibroblast growth factor 4	Possesses broad mitogenic and cell survival activities and is involved in a variety of biological processes, including embryonic development, cell growth, morphogenesis, and tissue repair	Unknown
	*Pex5*	Peroxisomal biogenesis factor 5	Targeting sequence involved in the specific transport of molecules for oxidation inside the peroxisome	Unknown
mmu-miR-877-5p	*Gpx1*	Glutathione peroxidase 1	Belongs to the glutathione peroxidase family, members of which catalyze the reduction of organic hydroperoxides and hydrogen peroxide by glutathione and, thereby, protects cells against oxidative damage	o
	*Aak1*	AP2 associated kinase 1	Regulates clathrin-mediated endocytosis by phosphorylating the AP2M1/mu2 subunit of the adaptor protein complex 2 (AP-2), which ensures high affinity binding of AP-2 to cargo membrane proteins during the initial stages of endocytosis	Unknown
	*Wnk3*	WNK lysine deficient protein kinase 3	Plays a role in the increase of cell survival in a caspase 3-dependent pathway as a positive regulator of the transcellular Ca2+ transport pathway	Unknown
	*Rab26*	RAB26, member RAS oncogene family	Important regulators of vesicular fusion and trafficking. The RAB family of small G proteins regulates intercellular vesicle trafficking, including exocytosis, endocytosis, and recycling	Unknown
	*Sult1e1*	Sulfotransferase family 1E, member 1	Catalyze the sulfate conjugation of many hormones, neurotransmitters, drugs, and xenobiotic compounds	Unknown
mmu-miR-149-3p	*Apoc2*	Apolipoprotein C-II	Extracellular region or secreted. Hydrolyzes triglycerides and thus provides free fatty acids for cells	o
	*Rab11fip5*	Rab11 family interacting protein 5	Rab effector involved in protein trafficking from apical recycling endosomes to the apical plasma membrane	Unknown
	*Leng8*	Leukocyte receptor cluster member 8	Predicted using known transcription factor binding site motifs	Unknown
	*Galnt17*	Polypeptide *N*-acetylgalactosaminyl transferase 17	Serve a variety of structural and functional roles in membrane and secreted proteins	Unknown

These antioxidant factors were predicted by the representative miRNA identified in the 20 μg/mL and 5 μg/mL treatment groups. Other miRNA involved the physiological development of exosomes and binding of vacuoles, or the prediction of most of the receptor-generating. Most importantly, the exosomal miRNAs isolated from MSCs were differentially expressed upon treatment with 5 and 20 μg/mL of the positively charged NPs. These findings indicated that specific antioxidants or tissue regeneration factors may be obtained from MSC-derived exosomes by exposing the MSCs to different concentrations of NPs (Table 1, Additional file 1: Figure S6).

### Endocytic mechanism of PLGA-PEI PCS NPs on MSCs

The changes in the internalization mechanism of iron oxide-based NPs were also examined to determine the factors involved in exosome generation. The surface modification of NPs can affect their interactions with cellular molecules. Several studies have reported a link between cytotoxicity and intracellular capacity [20, 23]. The absorption process is important for the physicochemical properties of the core particles and coating. Surface charge is also involved in stimulating molecules inside the cells [24]. Furthermore, properties and particle size are important determinants of the internalization mechanism [25]. While clathrin-mediated endocytosis internalizes NPs 120 nm in size, caveolae-mediated endocytosis internalizes NPs that are 60 nm in size [25, 26]. Therefore, we used two types of inhibitors (Dynasore and Pitstop2) to inhibit these endocytic pathways.

**Fig. 4:**
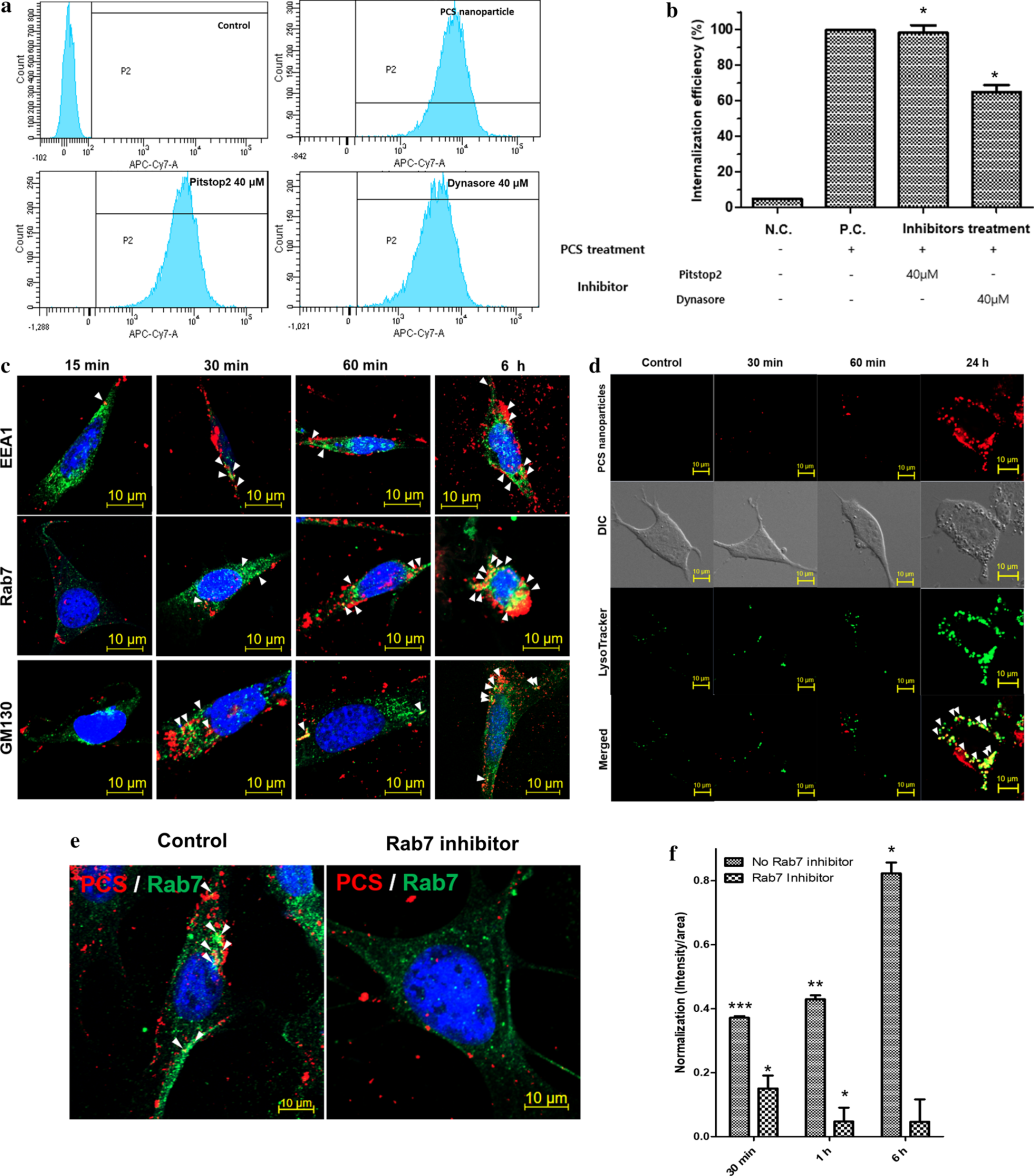
Analysis of PLGA-PEI PCS NPs internalization in MSCs. **a** Pitstop2 and Dynasore were used to analyze the mechanism of internalization. **b** FACS analysis revealed approximately 40% inhibition by Dynasore. N.C.: No treatment, P.C.: 5 μg/mL PLGA-PEI PCS NPs. **c** Immunofluorescence imaging analysis of the endocytic pathway of PCS NPs. The early endosome marker EEA1, the late endosome marker Rab7, and the Golgi apparatus marker GM130 were observed for 15 min, 30 min, 60 min, and 6 h after the MSCs were exposed to PCS NPs. Red florescence indicates PCS NPs and green florescence depicts EEA1 (first row), Rab7 (second row), and GM130 (third row). White arrows indicate the colocalization between NPs and cellular organelles. **d** Fluorescent images were taken after 30 min, 60 min, and 24 h to observe the colocalization between NPs and lysosomes. Red florescence indicates PCS NPs and green florescence represents LysoTracker Green detected in lysosomes. White arrows depict the merged areas between NPs and cellular organelles. **e** Characterization of endocytic pathway of PCS NPs using Rab7 inhibitor. No NPs are internalized, even after 6 h, in the presence of the inhibitor. PCS NPs are labelled red, while Rab7 is stained green. **f** PLGA-PEI PCS NPs are not detected inside the cells after 30 min, 1 h, and 6 h incubation periods with Rab7 inhibitor. Data are mean ± SD. *p < 0.05, **p < 0.05, ***p < 0.005

The two inhibitors did not completely inhibit the internalization of NPs. Approximately 60% of the NPs were internalized in response to the Dynasore treatment. However, the uptake of 5 μg/mL NPs was not inhibited when MSCs were treated with Pitstop2. The findings indicated that the PLGA-PEI PCS NPs were not internalized by clathrin-mediated endocytosis, and the approximately 40% inhibition by Dynasore was related to receptor-mediated endocytosis involving another mechanism (Fig. 4a, b). In addition, the examination of PLGA-PEI PCS NP internalization in viable MSCs (Additional file 1: Figure S7) revealed the inhibition of internalization of the NPs by both Dynasore and Pitstop2 (Additional file 1: Figure S8a, b). To observe the mechanism of PLGA-PEI PCS NP translocation in MSCs, we used cellular markers of organelles involved in endocytosis pathways [27, 28]. Early endosome antigen 1 (EEA1), Rab7, and GM130 were detected in MSCs treated with 5 μg/mL NPs for 15, 30 min, 60 min, and 6 h. As shown in Fig. 4c, NPs started to colocalize with EEA1 at 15 min. Since the NPs primarily followed the endocytic pathway, their transport could be initiated in the early endosomes. The cell internalization efficiency exceeded 80% after 30 min. Interestingly, the colocalization efficiency of Rab7, a late endosome and auto-phagosome marker, increased significantly after 6 h. The NPs appeared to be transported to the late endosomes following endosome maturation, and then to the larger organelles. NPs are considered to be transported to the auto-phagosomes and finally to the MVB [29]. NPs also appeared to colocalize with GM130, suggesting that some NPs were transferred to the Golgi. Several soluble and secretory molecules in the Golgi are involved in exocytosis. The imaging results suggested that they may be related to exocytosis since they overlap with NPs [9, 12]. Using LysoTracker, we observed that the PCS NPs were finally transported to lysosomes by the 24 h time point (Fig. 4d). However, it was not shown that PCS NPs were transported to the lysosome within 30 or 60 min. It is possible that the NPs could be degraded by lysosomal enzymes, whose activity would begin 6 h after the overlap with Rab7.

Here, immunofluorescence analysis revealed that Rab7 and PCS NPs displayed high levels of colocalization in the organelles at 6 h time point (Fig. 4c). Thus, we hypothesized that the transport of NPs could be blocked by the inhibition of Rab7 [30]. Rab7 expression was difficult to identify during the total reaction time of 6 h, and the merging efficiency decreased even when the cell area value was divided by the intensity value (Fig. 4e, f, Additional file 1: Figure S9). Interestingly, TEM revealed that the NPs in the majority of cells were trapped in vesicles that appeared to be early endosomes (Additional file 1: Figure S10). It was also difficult to find the traces of exosome expression on the cell surface even after a prolonged time. The findings from the investigation of the NP internalization mechanism with Rab7 support the evidence that a factor influences the expression of exosomes during their merger with the MVB or lysosome at the late endosome.

### Expression of autophagy-related genes

**Fig. 5:**
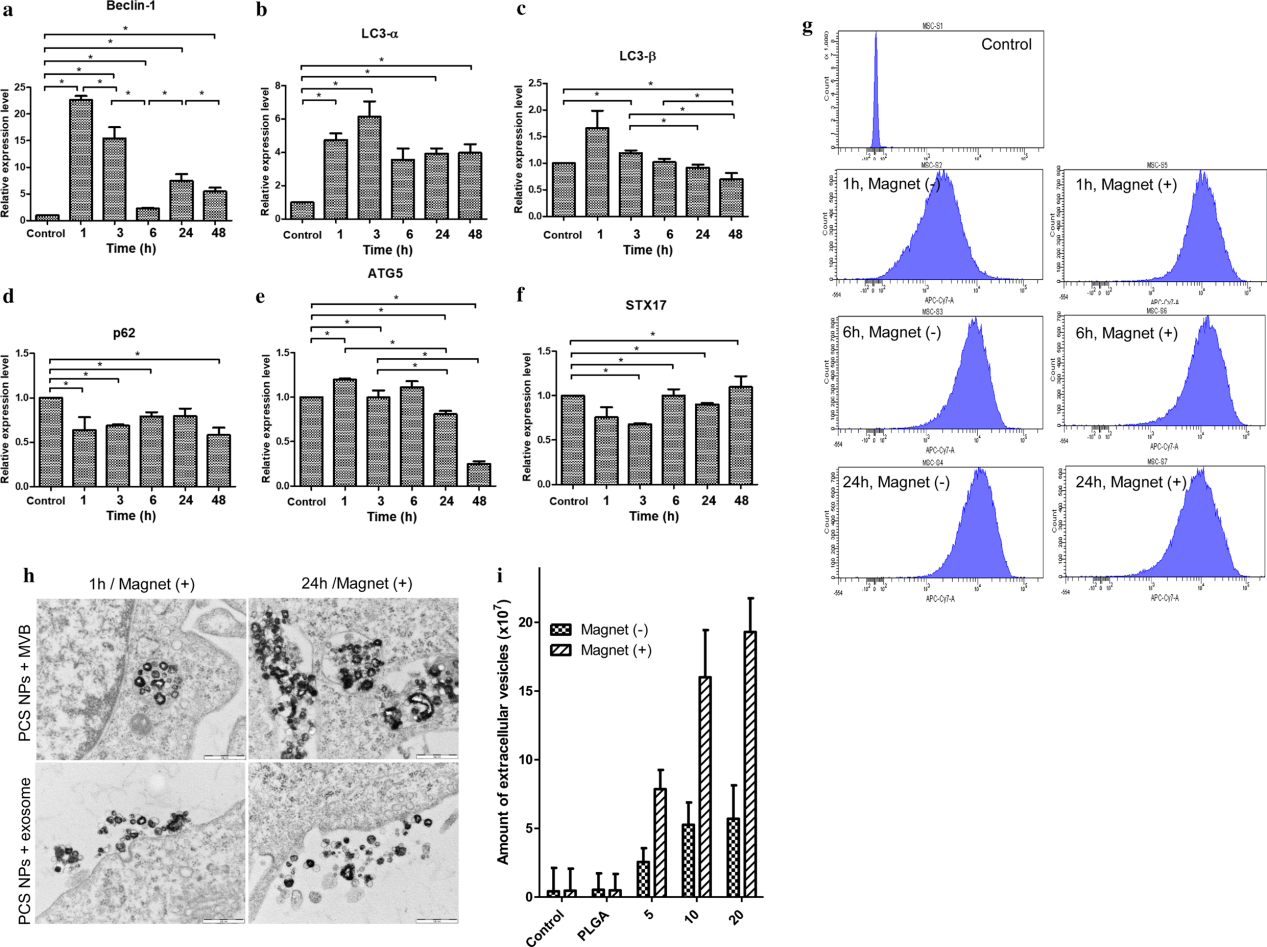
Gene expression levels of autophagy markers induced by NPs as measured by qRT-PCR. **a** Quantification of mRNA expressed after exposure to NPs for 1, 3, 6, 24, and 48 h. Immediately after NPs are added, Beclin-1 was expressed at 1 h and tended to decrease slowly. **b** *LC3-α* increased until 3 h with no difference after 6 h. **c** *LC3-β* increased at 1 h and gradually decreased thereafter. **d** *p62* expression levels decreased for the duration of the experiment. **e** *ATG5* displayed the highest expression in autophagosomes. **f** *STX17* showed higher after 6 h. The effect of magnetic and iron oxide-based PCS NPs on the development of exosomes. **g** After the incubation of the NPs with the MSCs, internalization by the magnet was confirmed by FACS. A large number of NPs were introduced into the cell by the magnet at 1 h. A similar number of NPs were introduced at 24 h in the absence of the magnet (−) as at 6 h with the magnet (+). **h** PLGA-PEI PCS NPs internalized in MVBs. It is possible that as the inflow of NPs increased, the generation of exosomes had also increased, resulting in the release of exosomes together with the undissolved NPs. When MSCs were treated with PCS NPs and magnets were active for 1 h, exosomes combined with NPs were generated. The amount of “PCS + exosome” increased at concentrations of 5, 10, and 20 μg/mL, with higher levels after 24 h. **i** Number of exosomes generated from cells, image analysis quantification. Without the magnet, the number of exosomes released in response to 5, 10, and 20 μg/mL PLGA-PEI PCS NPs was ~ 2.56 × 10^7^, 5.26 × 10^7^, and 5.7 × 10^7^, respectively. With the magnet, the numbers were ~ 7.86 × 10^7^, 1.6 × 10^8^, and 1.93 × 10^8^ exosomes released in response to 5, 10, and 20 μg/mL PCS NPs, respectively

To understand the mechanism of exosomal release, we decided to focus on the formation of MVBs. We have already identified the internalization pathway of NPs involved in the exosomal release, as well as the role of Rab7 in this process. This provided important evidence that MVB formation occurs in late endosomes and autophagosomes, the Rab7-positive organelles [31, 32]. We previously assessed whether autophagy is induced in MSCs by externally introduced NPs [33]. We measured the degree of change with time on the basis of the expression of the most relevant autophagy markers. The temporal expression of genes during exposure to NPs was determined using reverse transcription polymerase chain reaction (Fig. 5). Primers were designed to confirm the autophagy process (Additional file 1: Figure S11) [34–36]. The expression of these genes during 1, 3, 6, 24, and 48 h of exposure to NPs confirmed that autophagy was initiated during a short time period.

*Beclin-1* is expressed during the first phase of autophagy. A rapid increase in the expression of *Beclin-1* was evident after 1 h of NP exposure (Fig. 5a); although *Beclin-1* expression subsequently decreased over time, it remained significantly higher than those in the corresponding controls for each time point. When the control group was set to 1 h, the value increased by approximately 23 times, indicating that the expression was rapid, and autophagy was initiated. Microtubule-associated protein 1 light chain 3 (*LC3*) was examined next. *LC3*, which is generally converted from the alpha to beta form during the formation of the phagophore, was introduced into the membrane (Fig. 5b, c). When observed at different times, *LC3* displayed similar values between 1 and 3 h. The expression of *p62* was the highest at the 1 to 3 h time points, and gradually decreased thereafter (Fig. 5d). This gene expression pattern may reflect the influence of other mechanisms in addition to autophagy.

*ATG5* and *STX17* are genes that encode membrane proteins expressed at the end of autophagy when the autophagosome is combined with the autolysosome. The *ATG5* expression decreased slowly after 6 h (Fig. 5e). *STX17* expression also higher after 6 h (Fig. 5f). There was little difference in these factors in the formation of the autophagosome, and vesicle binding showed little difference. Therefore, PLGA-PEI PCS NPs induced degradation through the combination of lysosomes and autophagosomes.

### Alteration of the quantity of NPs in MSCs by magnetic attraction

The SPIONs used in this study have the capability to transport more NPs into cells. To increase the release of exosomes, MSCs were exposed to 5 μg/mL NPs for 1, 6, and 24 h in the absence or presence of magnets. We prepared a magnet 60 mm in size, which represents an intensity of approximately 0.6 T [21]. In the absence of the magnet, fluorescence-activated cell sorting revealed a gradual increase in the internalization rate of NPs over 24 h (Fig. 5g). In the absence of the magnet, the average mean intensity of NPs in MSCs was 2559.25 at 1 h, 9304.75 at 6 h, and 12,581.25 at 24 h. Detailed analysis of the intensity of NPs in the presence of a magnetic field for 1, 6, and 24 h is provided in Additional file 1: Figure S12.

In the presence of a magnetic field, NPs were increasingly internalized or adsorbed for 24 h compared to controls (Fig. 5h). In a novel observation, the combination of NPs and exosomes reduced the releaseof exosomes. These findings indicated that the expression of exosomes could be increased using positively charged NPs. NPs, in turn, stimulated the production of exosomes (Fig. 5h). In addition, to quantify the number of exosomes released by the cells, we performed image analysis (see formula in “Methods” section). The results of image analysis confirmed our previous observations that magnet application had increased the number of internalized NPs (Fig. 5i).

## Discussion

The aim of this study was to demonstrate the association between the generation of exosomes and the use of surface-modified NPs. Exosomes consist of double lipid membranes that encase 40–100 nm sized soluble proteins and RNA, and, therefore, are considered important for intercellular communication [8].

In this study, NPs developed through the combination of PLGA and PEI were used to increase the expression of exosomes in cells. The enhanced expression involved a positive surface charge and biocompatible PLGA [15]. Exosomes were expressed even when using commercially available positively charged PLGA-PEI NPs; however, the number of exosomes induced by FluidMag (+) was smaller than that induced by PLGA-PEI NPs (+). As in Fig. 2f, g, it was confirmed that the amount of exosomes released by the positively charged particles. The amount of exosomes by FluidMag NPs showed the lower a little bit than PLGA-PEI NPs. Therefore, it is important to examine the increased expression of exosomes in response to PLGA-PEI NPs. It has a mortality rate of approximately 40% by PCS NPs, and an increase in exosome markers may be related to cell death. However, in this study, we focused on research to prove the relationship of exosome release by NP. There are two important points demonstrated by this study. First, the study showed that the cell viability was about 90% at 5 µg/mL PCS NP concentration, and the number of exosomes increased compared to the control group. These results suggest that the exposure to NPs can sufficiently stimulate exosome release by the cells. Furthermore, as seen in Fig. 5, activation of the factors related to autophagy stimulated *Beclin-1* gene expression within 1 h. Second, the amount of exosome protein released into the medium was high despite the high toxicity of 40% in the 20 µg/mL PCS NP group. We discussed that MSCs received the highest toxicity as possible by NPs even if exosomes were released. Therefore, we believe that MVB formation and exocytosis, which contribute to exosome release, occurred before apoptosis; however, the induction of exosomal release by high toxicity requires further study. Additionally, the analysis of miRNAs that were increased within the exosomes also confirmed the expression of antioxidants and factors related to differentiation.

**Fig. 6:**
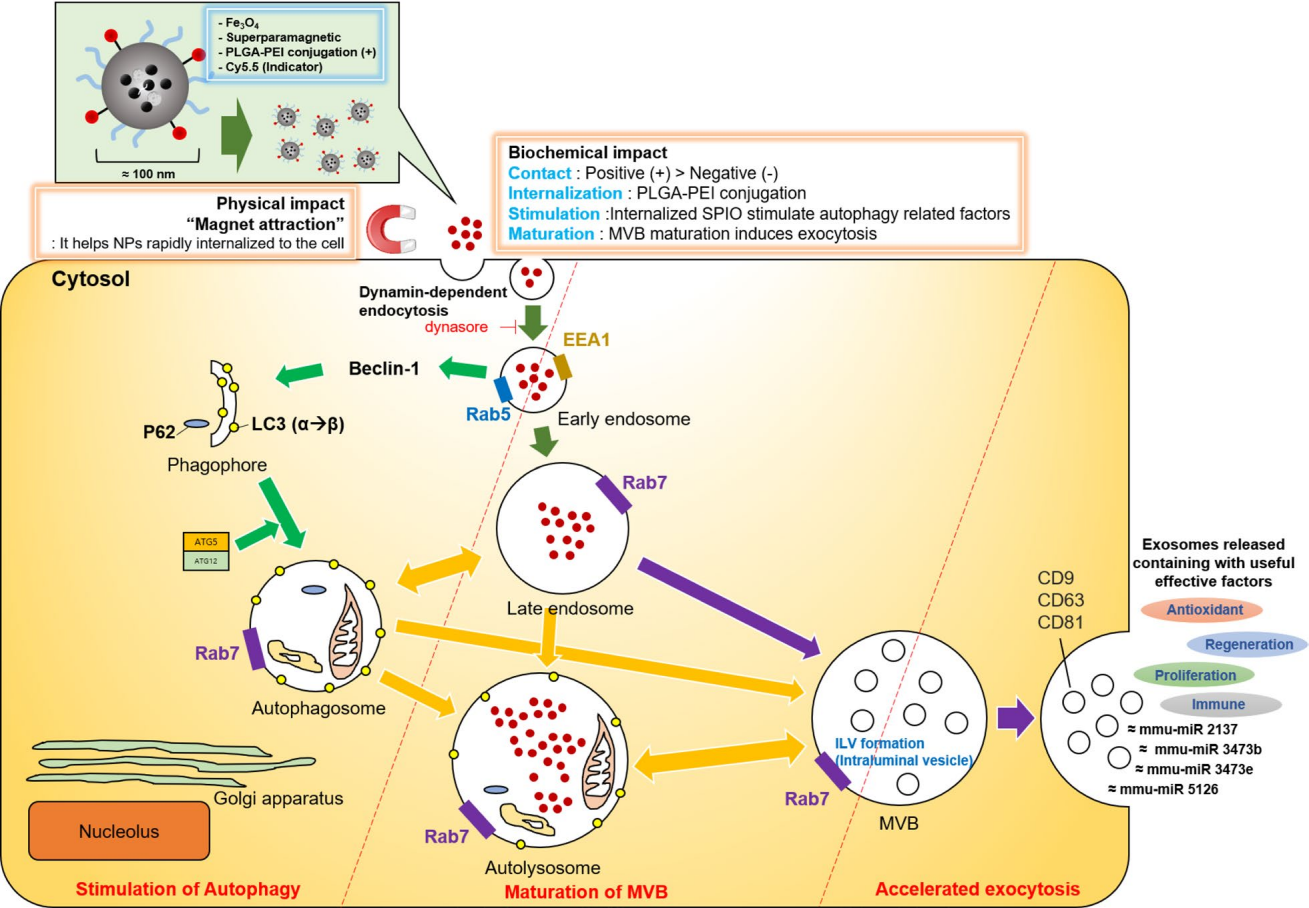
Schematic diagram of the internalization pathway of PLGA-PEI PCS NPs in MSCs. An accelerated path of exocytosis in response to the increased effect of internalization rate due to biochemical impact and physical impact was evident

The NPs presented in this study displayed different internalization pathways depending on their surface potential. We demonstrated that the mechanism of dynamin-dependent endocytosis of NPs in cells depended on the positively charged surface of PCS NPs. We explored the mechanism of overexpression of exosomes (Fig. 6). We found that the positively charged NPs stimulated Rab7 within 6 h (Fig. 4c), and when Rab7 was suppressed, the occurrence of exosomes was greatly reduced, so we revealed important evidence that Rab7 is an indicator of the pre-stage of exosomes. It is important to note that Rab7 is present in both the late endosomes and MVBs, but when the exocytic pathway is stimulated, MVB is the primary source of exosome release. Finally, we conclude that positively charged NPs can be induced by the overexpression of Rab7. It has been established that Rab7 is involved in MVB and autophagosome formation [29, 31]. Furthermore, Rab7 has been implicated in the functioning of class C core vacuole-endosome tethering and homotypic fusion and vacuole protein sorting tethering complexes and are converted systemically during endosomal maturation [37, 38]. Autophagy factors are also expressed when MSCs are treated with NPs. The expression of STX17, which is involved in autolysosome formation, was confirmed by the expression of Beclin-1 at 1 h. These results suggest that the expression of exosomes was due to the amplification of autophagy by the NPs introduced into the endosome, which resulted in an increase in autophagosomes and the accumulation of MVB in the cells.

We utilized iron oxide NPs to take advantage of magnetic attraction technology [19], which introduces NPs into cells by magnetism [17, 20]. We designed NPs with iron oxide to introduce more NPs and utilized PLGA and PEI to make it easier for NPs to bind to the cell membrane. The presence of a magnetic field increased NP internalization or adsorption compared to controls. This is a novel observation suggesting that NPs and exosomes could be combined to produce more exosomes without loss.

The findings presented here might be valuable in increasing the expression of exosomes using positively charged NPs. Our results clearly demonstrate that NPs stimulated the production of exosomes. In addition, we developed a formula that calculated the number of exosomes derived from cells using imaging analysis to determine the number of exosomes produced (see “Methods” section). This formula could be applied to increase the yield of exosomes in the laboratory; however, it would be difficult to apply it to clinical studies. Therefore, this study may influence future research through the expression of MSC-derived exosomes using positively charged NPs. Future studies of the mechanism by which exosomes are generated from stem cells by NPs are required, as treatment with NPs is thought to affect the composition and yield of exosomes. Through this study, we were able to confirm that a considerable amount of exosomes were released using positively charged nanoparticles, and we could expect to be able to study the generation of cell-derived exosomes using various types of positively charged nanoparticles. It is also expected that MSC-derived exosomes can be extracted from stem cells and used for therapeutic purposes, such as tissue regeneration or antioxidant efficacy.

## Conclusions

Currently, studies using stem cell-derived exosomes are gaining clinical interest. Therefore, research to improve the exosome yield is very important. In this study, we developed positively charged NPs based on iron oxide and PLGA, which can increase the incidence of exosomes (Fig. 6). We investigated the internalization mechanisms of the NPs and the factors affecting the increase of exosome yield. We anticipate that our findings will contribute to future developments of a NP-based technology to increase the yields of exosomes released from the MSCs.

## Methods

### PLGA-PEI and PLGA-Cy5.5 materials

PLGA (50:50, MW 38,000–54,000; Sigma-Aldrich, St. Louis, MO, USA) and PEI (MW ~ 25,000, M_n_ ~ 10,000; Sigma-Aldrich) were used to synthesize the PLGA-PEI copolymer. *N*,*N*ʹ-dicyclohexylcarbodiimide (DCC) and *N*-hydroxysuccinimide (NHS) were prepared as linkers between PLGA and PEI. PLGA (330 mg), PEI (113.67 mg), DCC (18.33 mg), and NHS (11 mg) were dissolved in 100 mL of dimethylsulfoxide (DMSO) solution and incubated for 24 h. After the completion of this reaction, the reaction mixture was dialyzed (MW cutoff = 10,000) with deionized water to remove excess DCC, NHS, and PEI. After dialysis, the polymer solution was freeze-dried to obtain the powdered sample.

To synthesize the PLGA-Cy5.5 fluorescence polymer, 330 mg of PLGA was dissolved in DMSO along with 60 mg of 1-ethyl-3-(3-dimethylaminopropyl) carbodiimide hydrochloride (EDC, Thermo Fisher Scientific, Waltham, MA, USA), 132 mg of NHS, and 9.9 mg of Cy5.5 (Cy5.5, red; Lumiprobe Co., Hannover, Germany), and incubated for 24 h. After the completion of this reaction, the reaction mixture was extensively dialyzed (MW cutoff = 10,000) with deionized water to remove excess EDC, NHS, and Cy5.5. The resulting PLGA-Cy5.5 polymer was freeze-dried to obtain the powdered sample [15].

### Synthesis of polymeric clustered SPIO (PCS) NPs

To manufacture the positively charged surface-modified NPs (PLGA-PEI PCS NPs), 0.1 mg of the PLGA-PEI copolymer and 0.4 mg of PLGA-Cy5.5 were dissolved in 0.1 mL DMSO. Iron oxide (II, III) magnetic NPs solution (0.1 mL containing 5 mg/mL) was added dropwise to 3 mL of deionized water. The vial was vortexed for 5 min followed by 3 min of sonication. The solution mixture was stirred at room temperature for 6 h. At the end of the process, the obtained NPs were further purified by ultra-centrifugation and stored at 4 °C until further use.

PLGA and SPIO were used to manufacture negatively charged, surface-modified NPs. EDC and NHS were prepared as a linker between modified NPs and the Cy5.5 fluorescence molecule that was synthesized for the detection of NPs in the cells. Specifically, these PCS NPs, otherwise known as PLGA-Cy5.5, were synthesized by EDC-NHS coupling. For this, 330 mg of PLGA was dissolved in DMSO along with 60 mg of EDC, 132 mg of NHS, and 9.9 mg of Cy5.5, and incubated for 24 h. After the completion of this reaction, the reaction mixture was extensively dialyzed (MW cutoff = 10,000) with deionized water to remove excess EDC, NHS, and Cy5.5 by purification, therefore, are not expected to cause any side effects in this study. The resulting PLGA-Cy5.5 particles were freeze-dried to obtain the powdered samples. We used 100% PEI to create positively charged NPs and 100% PLGA to produce negatively charged NPs. In addition, positively charged NPs have also been made using the 8:2 ratio of PLGA:PEI.

### Characterization of NPs

The PCS NPs with a polymeric shell consisting of 10 nm diameter SPIOs in the core were prepared using an oil-in-water emulsion method. In a typical experiment, 1 mg of PLGA-Cy5.5 was dissolved in acetonitrile at 25 °C. SPIO (0.1 mL containing 5 mg/mL) was added dropwise to 3 mL of deionized water. Next, the vial was vortexed for 5 min followed by 3 min of sonication. The solution mixture was stirred at room temperature for 6 h. At the end of the process, the obtained NPs were further purified by ultra-centrifugation and stored at 4 °C until further use. For comparison, we purchased positively charged (FluidMAG-Chitosan, cat. 4118-1; Chemicell GmbH, Berlin, Germany) and negatively charged (FluidMAG-CMX, cat. 4106-1; Chemicell GmbH) NPs 100 nm in size. The surface charge was reported to be + 50 mV for FluidMAG-Chitosan [14] and − 14 mV for FluidMAG-CMX [31].

The size and surface zeta potential of the NPs were obtained by dynamic light scattering using a Zetasizer-ZS90 apparatus (Malvern Instruments, Malvern, UK). The morphology of the particles was characterized by scanning electron microscopy (SEM) using a JSM-7100F instrument (JEOL, Tokyo, Japan). The samples for SEM were prepared by adding a droplet of the NP suspension (2 μL) to a polished silicon wafer. After drying the droplet at room temperature for 4 h, the sample was coated with platinum and imaged using SEM. To further evaluate the internal SPIO core, TEM measurements were performed. The samples for TEM were prepared by the drop casting method over a carbon grid as previously described [15, 25].

### Cell culture

MSCs (cat. MUBMX-01001) and green fluorescent protein (GFP)-labeled MSCs (cat. MUBMX-01101) were purchased from Cyagen (Santa Clara, CA, USA). The cells were cultured in alpha-MEM containing 10% fetal bovine serum and 1× antibiotics. The medium was changed every 2 days after washing with Dulbecco's phosphate-buffered saline in a humidified atmosphere of 5% CO_2_.

### Flow cytometry analysis

GFP-labeled MSCs were plated in 60 mm culture dishes at 1 × 10^5^ cells/well. After 24 h incubation period with PCS NPs, the cells were washed twice with PBS. Next, samples were analyzed by flow cytometry using a FACS Aria3 flow cytometer (Becton Dickinson, San Jose, CA, USA). The data were analyzed using the FACS Diva software.

### Viability assay

Cell viability was measured by counting viable cells using the EZ-3000 CCK kit (Dogen, Seoul, Korea) (CCK-8) according to the manufacturer’s instructions. Briefly, the MSCs were seeded into 96-well plates (5 × 10^2^ cells/well) and incubated at 37 °C in 5% CO_2_ for 24 h. The next day, the culture medium was replaced with 200 μL of fresh medium containing different concentrations of PCS NPs. After incubation for 24 h, 20 μL of CCK-8 was added and the cells were protected from light. The absorbance values at 450 nm were measured with an enzyme-linked immunosorbent assay reader after 4 h of incubation.

### Fluorescence analysis

MSCs and GFP-labeled MSCs were seeded (5 × 10^3^ cells/well) into 4-well cell culture plates. After 24 h of incubation, the culture medium was replaced with fresh medium and NPs were added. After incubation for 24 h with NPs, the MSCs were washed twice with PBS, fixed in 10% paraformaldehyde, and stained with 4,6-diamidino-2-phenylindole (DAPI). Even though the NPs labeled with Cy5.5 (red) were easy to detect in cells, the cellular organelles were also stained with specific antibodies. The following primary antibodies were used: anti-EEA1 (ab2900; Abcam, Cambridge, MA, USA) for early endosomes, anti-Rab7 (Aab126712; Abcam) for late-endosomes, and anti-GM130 (ab169276; Abcam) for the Golgi apparatus. To localize the PCS NPs in cellular organelles, goat anti-rabbit IgG H&L (Alexa Fluor 488, green) secondary antibody (ab150077; Abcam) was used. In addition, Lyso-Tracker green DND-26 (cat. L7526; Life Technology, Carlsbad, CA, USA) was also used to confirm the localization of the NPs in the cells. All samples were observed by confocal microscopy (Carl Zeiss Microscopy GmbH, Jena, Germany) and images were analyzed using ZEN lite ver. 2.3 software.

### Inhibitors

Two inhibitors of NP internalization were used: Dynasore (ab120192, Abcam) and Pitstop2 (SML-1169, Sigma-Aldrich). Both inhibitors were used at a concentration of 40 μM/dish and they remained active 3 h prior to NP treatment. Dynasore inhibits dynamin, an important factor in receptor-mediated endocytosis, while Pitstop2 blocks clathrin-mediated endocytosis in the receptor-mediated endocytosis pathway. Both inhibitors were added to MSCs for 30 min before treatment with positively charged PCS NPs. CID 1067700 (cat. 2184, Axon Medchem, Groningen, Netherlands) was used to block Rab7 by competitive inhibition.

### TEM

Cells were fixed in 2.5% glutaraldehyde for 2 h at 4 °C and the specimens were solidified on 2% agar. Samples were post-fixed in 1% osmium tetroxide after being washed with 0.1 M cacodylate buffer. Dehydration was performed in a stepwise manner using 50% to 100% ethanol gradient. Next, the samples were embedded in Epon resin. Ultrathin sections were cut using an ultra-microtome and stained with uranyl acetate and lead citrate. TEM was performed at 63 kV.

### Preparation of exosomes

Exosomes from the medium of cultured MSCs were isolated using Total Exosome Isolation reagent (4478359; Invitrogen, Carlsbad, CA, USA), strictly according to the manufacturer’s instructions. PBS was used to resuspend the purified exosomes. Exosomes were stored at − 80 °C for long-term preservation or at − 20 °C for short-term preservation. Exosomal proteins were isolated using lysis buffer containing 0.1% Triton X-100 in PBS and a protease inhibitor cocktail.

### Real-time reverse transcription polymerase chain reaction (qRT-PCR)

To prepare mRNA samples, a total volume of 10 µL, including 2 µL of mRNA and 8 µL of reverse transcriptase mix, was used. The mixture included 1 µL of 10× enzyme mix, 2 µL of 5× enzyme reaction buffer, and 5 µL of nuclease-free water. According to the manufacturer’s protocol for the cDNA synthesis kit (TOYOBO, Osaka, Japan), samples were incubated at 42 °C for 60 min and deactivated at 95 °C for 5 min. Next, cDNA was diluted 1:10 with 90 µL nuclease-free water for miRNA qRT-PCR.

qRT-PCR was performed using Applied Biosystems 7900 thermal cycler (Applied Biosystems, Foster City, CA, USA). Samples were subjected to reverse transcription using the SYBR Select master mix (Applied Biosystems) following the manufacturer’s protocol. The following primers were used for sequencing: 18S rRNA, forward: 5′- CTA ACC CGT TGA ACC CCA TT—3′ and reverse: 5′- CCA TCC AAT CGG TAC TAG CG—3′; Beclin-1, forward: 5′- GGA GCT GGA AGA TGT GGA AA—3′ and reverse: 5′—ACT CCA GCT GCC TTT TA—3′; LC3-α, 5′-GCC TGT CCT GGA TAA GAC CA—3′ and reverse: 5′- GGT TGA CCA GCA GGA AGA AG—3′; LC3-β, 5′- CCG AGA CCT TCA AGC AG—3′ and reverse: 5′- CTC CCC CTT GTA TCG CTC TAT—3′; p62, 5′- GTC TTG GGG AAG GGT TCA AT—3′ and reverse: 5′-GGG GGT CCA AAG ACT TCA AT—3′; ATG5, 5′- ATA TGA AGG CAC ACC CCT GA—3′ and reverse: 5′—CAT CCT TGG ATG GAC AGT GTA G—3′; STX17, 5′- GAT CCC AAC AGA CCT GGA GA- 3′ and reverse: 5′- GAT ATT GGA GCG CAG TTG CT—3′. The total reaction volume of 10 µL included 1 µL cDNA, 5 µL of premix, 1 µL of 10 pmol forward and reverse primers, and 3 µL of nuclease-free water. qPCR cycle conditions and steps were as follows: pre-amplification at 95 °C for 3 min, 40 cycles of 95 °C for 10 s, 60 °C for 60 s, and melting curve analysis. All qPCR assays were performed in duplicate. The mRNA expression levels were normalized using 18S rRNA.

### miRNA analysis in exosomes

RNA samples were obtained from the medium treated with 5 or 20 μg/mL PCS NP for 24 h. The concentration of the sample was confirmed through quality control analysis and was confirmed to be between 0.17 and 0.26 ng/μL. To check the purity and quantity of RNA, a NanoDrop spectrophotometer was used to measure the absorbance at 260 nm and 280 nm. All raw data were extracted automatically in the Affymetrix data extraction protocol using the Affymetrix GeneChip^®^ Command Console^®^ Software (AGCC). The CEL files were imported, miRNA levels were normalized using the RMA algorithm, the detection above background p-values were calculated for all data, and the results were exported using the Affymetrix^®^ Power Tools (APT) Software.

Array data were filtered using species-specific annotated probes. The comparative analysis between the test and control samples was carried out using fold change. All statistical testing and visualization of differentially expressed genes were conducted using R statistical language 3.3.3 (https://www.r-project.org/).

### Magnetic attraction

MSCs were sub-cultured, plated in a 60 mm dish at 2 × 10^5^ cells/dish, and incubated for 24 h. The next day, the NPs were added and a magnet of the same size as the plate was immediately placed under the bottom of the plate. The cells were incubated for 1, 6, or 24 h, and then washed with PBS. Cells were detached using trypsin and the mean intensity value was measured using FACS in a round vial. For TEM, electron microscopy images were analyzed as described below.

### Imaging analysis quantification of exosome amounts

To quantify the number of exosomes using image analysis, the images of cells expressing exosomes with a size of 50 to 150 nm were obtained. Equations (1) and (2) were created to quantify the number of exosomes. The value definition within the equation is as follows:

1. n = number of images, E = average number of released exosomes,
2. C = cell × dilution ratio
  1 $$\mathrm{Exosome amount }\left(\mathrm{EA}\right)= \frac{E}{n}\times C$$
3. ΔT = total amount of time, k = time change, t = time,
4. A = exosome amount/single cell
  2 $$\frac{EA}{\Delta T}=\sum_{0\to k}^{t}{A}_{k} , A=\frac{E}{n}\times C$$

### Statistical analyses

qPCR results were analyzed to compare the mRNA levels of autophagy markers. The Mann–Whitney U test and receiver operating characteristic curve analysis were performed using SPSS version 24.0 (IBM, Armonk, NY, USA). All graphs were plotted using GraphPad PRISM version 5.0 (GraphPad Inc., La Jolla, CA, USA). The sensitivity and specificity of the statistically significant mRNAs were analyzed using GraphPad PRISM version 5.0. p-values < 0.05 were considered statistically significant.

## Supplementary information

**Additional file 1: Figures S1–S12.** Provide the summary of the results characterizing the nanoparticles, electron microscopy images of exosomes, list of primers. The additional information includes detailed experimental materials and synthesis strategies, methods for miRNA analysis, and formulae for exosome quantification.

**Additional file 2.** Movie of PLGA-PEI PCS NP internalization. The additional information includes detailed experimental materials and synthesis strategies, methods for miRNA analysis, and formulae for exosome quantification.

## Data Availability

All data analyzed during this study are included in this published article (Additional files 1 and 2).

## Abbreviations

DCC: *N*,*Nʹ*-Dicyclohexylcarbodiimide
EVs: Extracellular vesicles
MSC: Mesenchymal stem cells
MVB: Multiple vesicle body
NHS: *N*-Hydroxysuccinimide
NPs: Nanoparticles
PCS: Polymeric clustered superparamagnetic
PEI: Polyethylenimine
PLGA: Poly-d,l-lactide-co-glycolide
SPIONs: Superparamagnetic iron oxide NPs
