# Supplementary material for: Improvement of stem cell-derived exosome release efficiency by surface-modified nanoparticles
Source: J Nanobiotechnology. 2020 Dec 7;18:178. doi: 10.1186/s12951-020-00739-7 (PMC7720507; doi:10.1186/s12951-020-00739-7)
Supplement: Supplementary file 1 — Additional file 1: Figures S1–S12. Provide the summary of the results characterizing the nanoparticles, electron microscopy images of exosomes, list of primers. The additional information includes detailed experimental materials and synthesis strategies, methods for miRNA analysis, and formulae for exosome quantification. [file 12951_2020_739_MOESM1_ESM.docx]

**Additional file 1**

**Improvement of Stem cell-derived Exosome Release Efficiency
by Surface Modified Nanoparticle**

Dong Jun Park^1,2^, Wan Su Yun^3^, Woo Cheol Kim^c^, Jeong-Eun Park^1,2^, Su Hoon Lee^1,2^, Sunmok Ha^4^, Jin Sil Choi^1,2^, Jaehong Key^3^ and Young Joon Seo^1,2*^

^1^ Department of Otorhinolaryngology, Yonsei University Wonju College of Medicine, 20 Ilsan-ro, Wonju, Gangwon-do 26426, Wonju, South Korea

^2^ Research Institute of Hearing Enhancement, Yonsei University Wonju College of Medicine, Wonju, South Korea

^3^ Department of Biomedical Engineering, Yonsei University, Wonju, South Korea

^4^ Department of Biomedical Laboratory Science, College of Health Sciences, Yonsei University, Wonju, Republic of Korea

*Correspondence:

**Young Joon Seo, MD, PhD**

Department of Otorhinolaryngology, Yonsei University Wonju College of Medicine, 20 Ilsan-ro, Wonju, Gangwon-do 26426, South Korea

[okas2000@hanmail.net](mailto:okas2000@hanmail.net)

Tel.: +82-33-741-0644


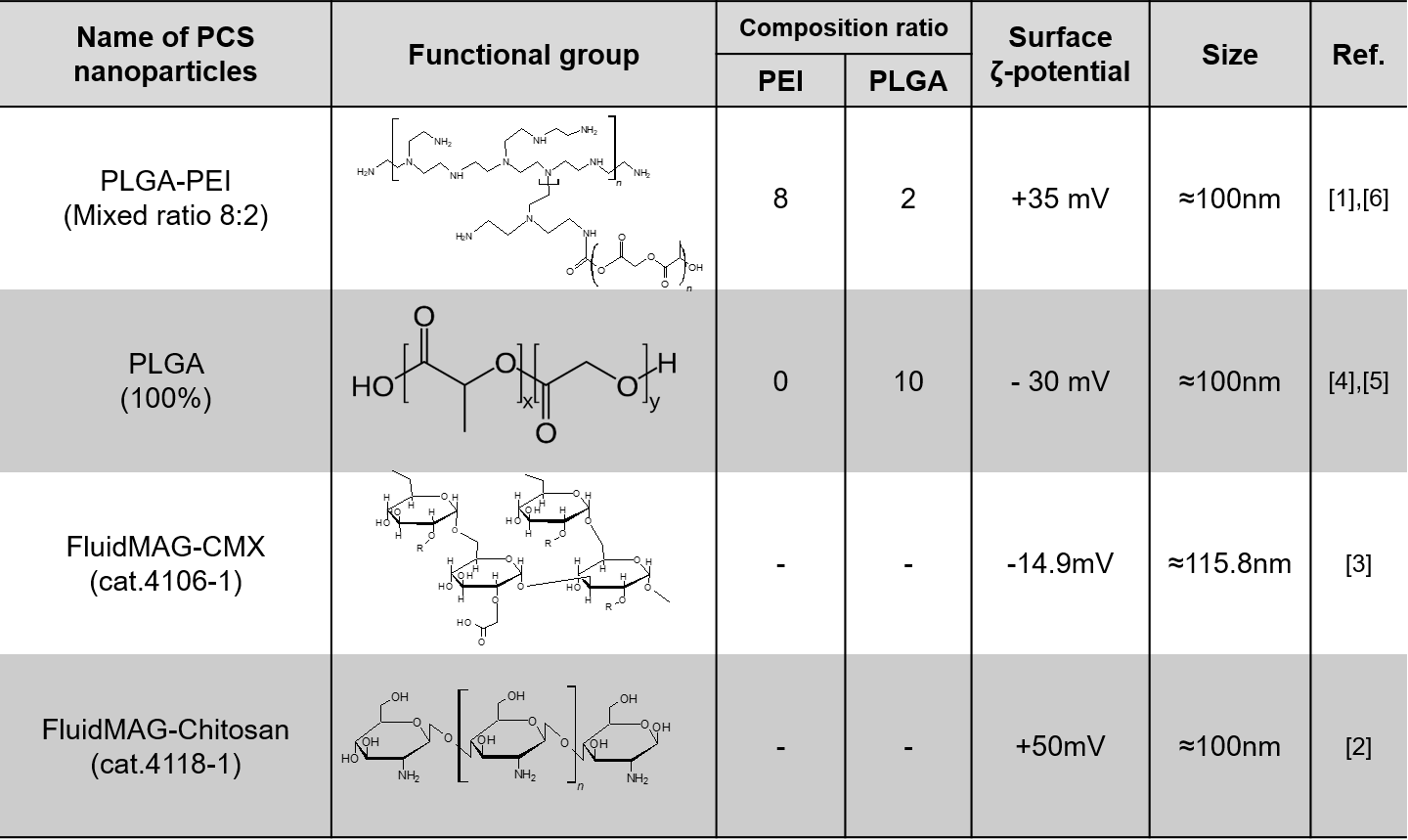


**Figure S1** The three surface-modified nanoparticles and two purchased nanoparticles used in the study. All nanoparticles are about 100 nm with different surface charges. The surface ζ-potential of PLGA-PEI (ratio 8:2) and FluidMAG-Chitosan are positively charged NPs. Whereas, PLGA (100%) and FluidMAG-CMX showed a negative surface charged NPs.

**
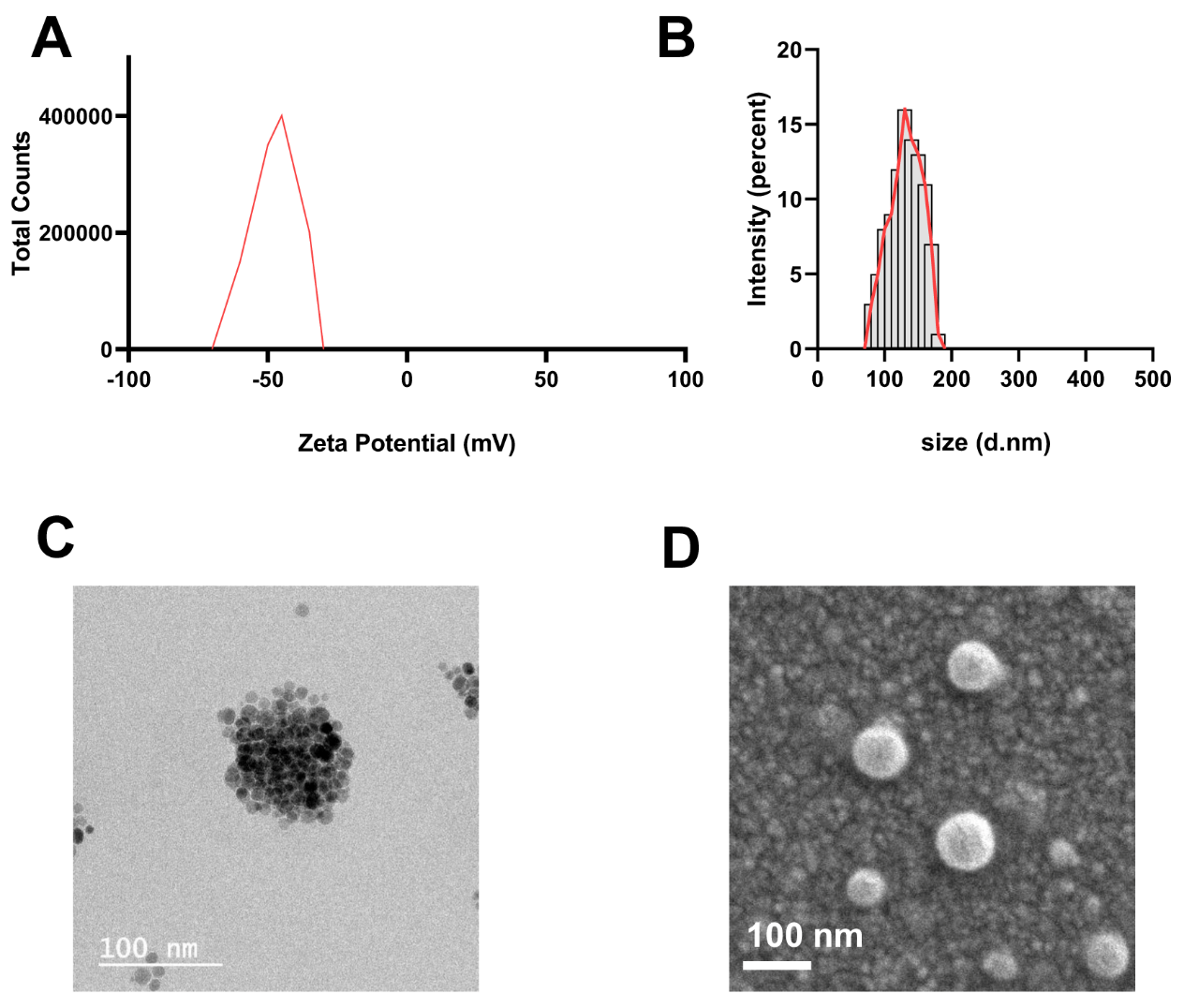
**

**Figure S2** Properties of negative charged PCS NPs. (a) Schematic of PCS NPs. (b) Size. (c) ζ-potential. (d) TEM image. e SEM image.

**
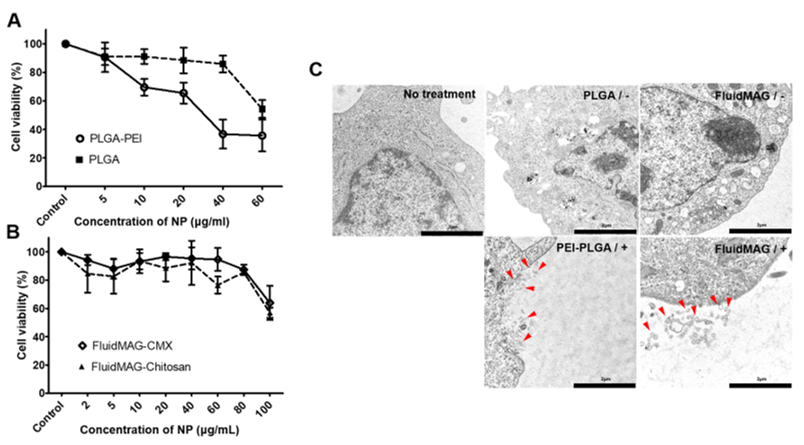
**

**Figure S3** Establishment of mesenchymal stem cell (MSC)-derived exosomes and concentration conditions of the various nanoparticles (NPs). (A) Cell viability of MSCs incubated with PLGA-PEI and PLGA PCS NPs at different concentrations for 24 h. (B) Cell viability of MSCs incubated with FluidMag-CMX and FluidMag-Chitosan at different concentrations for 24 h. (C) Exosomes released from MSCs exposed to NPs with different surface charges.

**
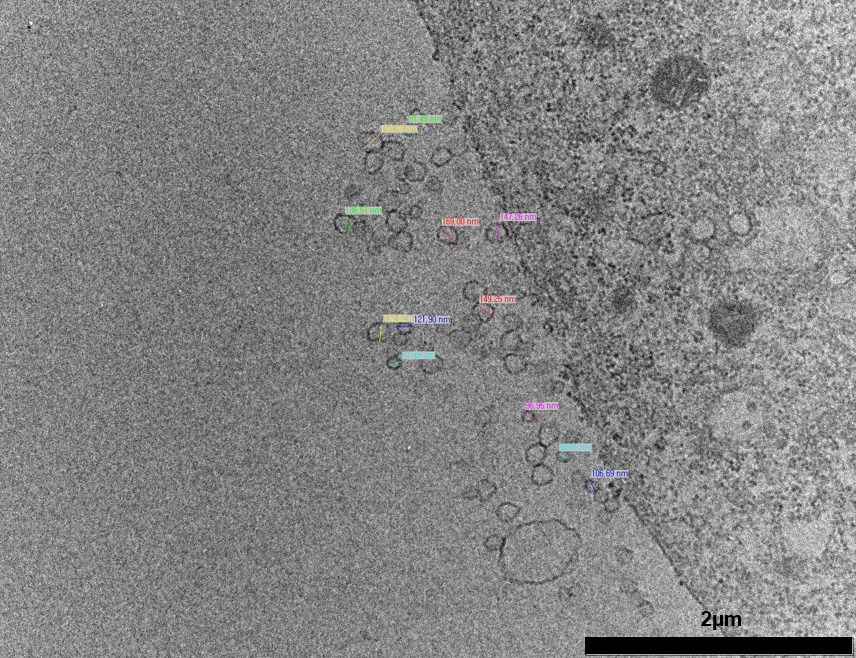
.**

**Figure S4** Diameter of MSC-derived exosomes measured by image analysis. To determine the overexpression of exosomes by our NPs, the exosomes were purified using the exosome isolation kit after treating the MSCs with PCS-NPs for 24 h. Purified exosomal total RNA was isolated, and the samples were quantified as described in the methods section. The exosomes released from MSCs were randomly measured. The sizes were 91.49, 121.9, 91.49, 96.95, 98.01, 106.69, 132.32, 147.25 nm, and the average was 110.76 ± 20.8 nm. The cells were fixed in 2.5 % glutaraldehyde for 2 h at 4 ºC, and the specimens were left to solidify in 2 % agar. Samples were post-fixed in 1% osmium tetroxide (OsO_4_) after being washed in 0.1 M cacodylate buffer. The dehydration steps were performed using 50 to 100 % ethanol and embedded in Epon resin. Ultrathin sections were cut using an ultra-microtome and stained using uranyl acetate and lead citrate. After observation using a transmission electron microscope, all samples were measured by the calculate module of imaging analysis. The diameter of the exosomes length was measured to represent the mean and to calculate the error.

**
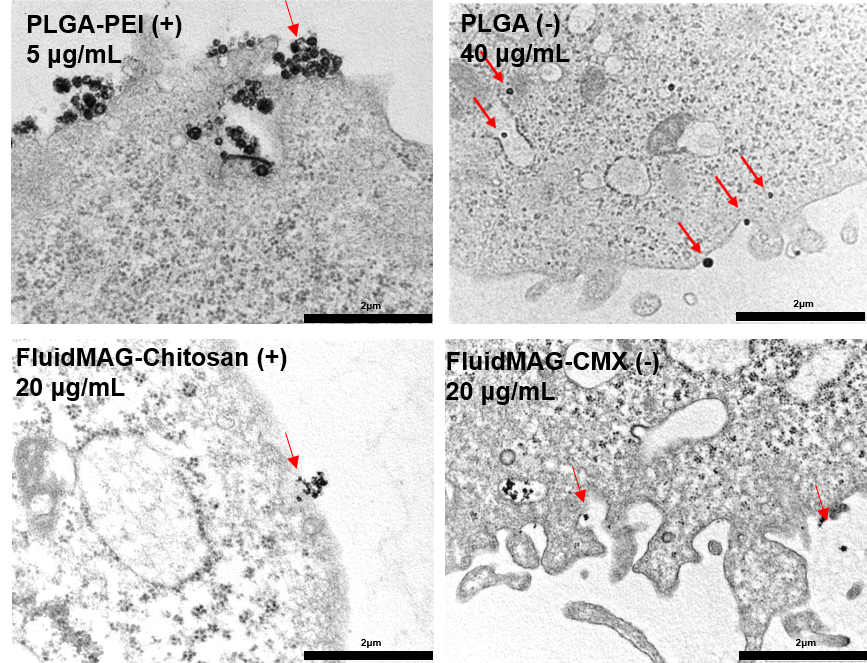
**

**Figure S5** The various images of internalization of nanoparticles on the MSC surface. PLGA-PEI and FluidMAG-Chitosan were used as positively charged PCS NPs. PLGA and FuildMAG-CMX were used negatively charged PCS NPs. The MSCs were treated with non-toxic concentrations of nanoparticles.

**
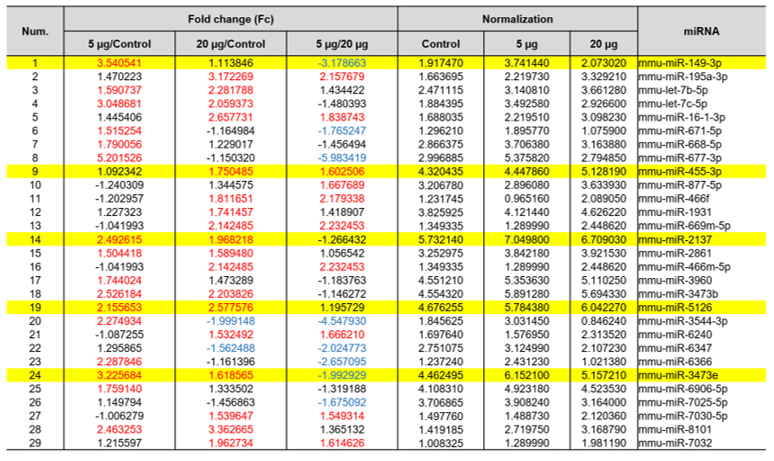
**

**Figure S6** List of analyzed miRNA of MSC-derived exosomes after treatment with 5 and 20 µg of PCS nanoparticles. A total of 29 miRNA were analyzed and normalized by comparing control, 5, and 20 µg samples. In addition, we compared control-5 µg, control-20 µg, and 5 µg-20 µg. Fold change increases of more than 1.5 times are shown in red, and decreases of more than 1.5 times are in blue. The shaded area in yellow represents the analyzed miRNA.

**miRNA analysis.** As a result of analyzing the four miRNAs commonly increased in cells exposed to NPs, 178 mRNAs were predicted in mmu-miR-2137. We selected targets with a matching score over 90% in the predicted target database that were involved in antioxidant efficacy and anti-apoptosis, according to previous reports [7]. They included glutathione peroxidase 4 (Gpx4), Golgi SNAP receptor complex member 1 (Gosr1), Ras-related protein Rab-4A (Rab4A), paired related homeobox 1 (Prrx1), and peroxisomal biogenesis factor 26 (Pex26). In more detail, Gpx4 is known to play a key role in protecting cells from oxidative damage by preventing membrane lipid peroxidation [8]. Gosr1 is known to regulate intracellular ROS levels via inhibition of p38 MAPK (MAPK11, MAPK12, MAPK13, and MAPK14) [9]. RAB4A is related to protein transport processes; it plays a role in vesicular traffic and mediates VEGFR2 endosomal trafficking to enhance VEGFR2 signaling [10]. The other two mRNAs were not reported to be exosomal target mRNAs; however, they could be inferred to be target RNAs. Prrx1 is known to enhance DNA-binding activity and the induction of genes by growth and differentiation factors [11]. Pex26 is related to peroxisome biogenesis factor 1 (also known as PEX1) [12]. The five predicted target mRNAs were involved in antioxidants or the conjugation of vacuoles.

Next, mmu-miR-3473b was analyzed, and it included 613 predicted target mRNAs, including Ring finger and SPRY domain containing 1 (Rspry1), Eph receptor B2 (Ephb2), complexin 2 (Cplx2), interleukin 7 (IL-7), and RAB11 family interacting protein 1 (class I) (Rab11fip1). These mRNAs were primarily involved in stimulating the differentiation of multipotent hematopoietic stem cells or encoded binding receptors and were associated with exosome release. Cplx2 refers to a one of a small set of eukaryotic cytoplasmic neuronal proteins that binds to the SNARE protein complex (SNARE pin) with a high affinity. It acts as both an inhibitor and a facilitator of synaptic vesicle fusion and neurotransmitter release [13]. IL-7 stimulates the differentiation of multipotent (pluripotent) hematopoietic stem cells into lymphoid progenitor cells [14]. The function of Rab11fip1, which encodes the interaction of Rab11 and FIP, is unknown; however, it is involved in the formation of a targeting complex that recruits a group of proteins involved in membrane transport to organelles. Currently, several Rab11-FIP complex-binding proteins have been identified that regulate distinct membrane traffic pathways. Rab11 is also involved in controlling membrane trafficking with the endosomal recycling process along the phagocytic pathway and in phagocytosis [15, 16].

Mmu-miR-3473e was also analyzed and predicted 613 target mRNAs. Of these, we selected four predicted mRNAs, including ST6-N-acetylgalactosaminide alpha-2,6-sialyltransferase 6 (St6galnac6), adenylate cyclase 1 (Adcy1), serine/threonine-protein kinase WNK2 (Wnk2), and signal-induced proliferation-associated 1-like protein 3 (Sipa1l3). Most of these were involved in endosomal and intracellular binding. Importantly, a case was found involving mRNA-encoding proteins that affect cell differentiation. St6galnac6 is related to the modification of ceramides on the cell surface to alter cell-cell or cell-extracellular matrix interactions. Adcy1, Wnk2, and Sipa1l3 play important roles in the regulation of homeostasis, cell survival, and proliferation [17-19], and these are involved in hearing loss and cell structure formation that may be caused by a defect in the gene. Therefore, these findings may be useful for the application of therapeutic efficacy.

Finally, mmu-miR-5126 identified four predicted target mRNAs out of 10 candidates, which included paired box 2 (Pax2), V-type proton ATPase subunit C 2 (Atp6v1c2), CD4 molecules (CD4), and autoimmune regulator (Aire). These primarily have anti-immune roles and serve to encode proteins involved in membrane construction and physiological responses. One of them, AIRE, expressed by a distinct bone marrow-derived population, induces self-tolerance through a mechanism that does not require regulatory T-cells and is resistant to innate inflammatory stimuli.[20]. However, CD4 and Atp6v1c3 were reported in the exosomal miRNA database.

A comparison of the 5 and 20 μg/mL groups showed two miRNAs to be increased (mmu-miR-877-5p and mmu-miR-455-3p), and three miRNAs to be decreased (mmu-miR-7025-5p, mmu-miR-3473e, and 677-3p) (Figure 2E). We selected mmu-miR-455-3p, which predicted 393 target genes, including TELO2-interacting protein 1 homolog (Tti1), hexokinase 3 (Hk3), secretion associated Ras related GTPase 1A (Sar1a), fibroblast growth factor 4 (Fgf4), and peroxisomal biogenesis factor 5 (Pex5). Tti1, Hk3, and Sar1a were reported in the exosomal miRNA database, but Fgf4 and Pex5 were not. HK3 also functions to protect the cell against apoptosis. Overexpression of HK3 results in increased ATP levels, decreased ROS production, an attenuated reduction in the mitochondrial membrane potential, and enhanced mitochondrial biogenesis [21]. Sar1a is involved in membrane trafficking. It is a monomeric small GTPase found in COPI vesicles and protects against oxidative stress and inflammation. It also regulates the assembly and disassembly of COPII coats [22]. Mmu-miR-149-3p was analyzed to predict 170 target mRNAs with the highest fold change value in the control and 5 μg/mL groups. As a result, it did not appear much in the exosome database, and only apolipoprotein C-II (APO2) was confirmed. This miRNA encodes AP2 and is known to be a factor involved in the secretion of vesicles. It also known to hydrolyze triglycerides and thus provides free fatty acids for cells [23].

**
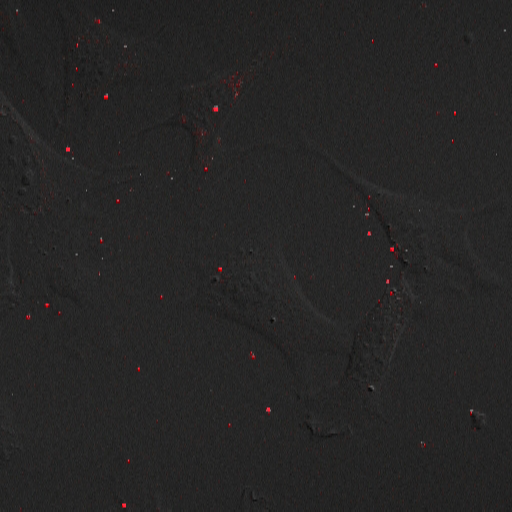
**

**Figure S7** Live cell image of PCS nanoparticle internalization into MSCs. The MSCs were seeded onto confocal dishes at a density of 5×10^3^ cells/well. After 24 h of incubation, the culture media was replaced with fresh media and treated with PCS nanoparticles. The MSCs were washed twice with PBS and observed on Cy5.5 (red) immediately. After treatment with 5 µg PCS nanoparticles, the cells were imaged for 2 h to record the initial stage of internalization. As a result, we confirmed that the nanoparticles were slowly introduced into the interior after they adhered to the surface. (Attached movie: Additional file 2)

**
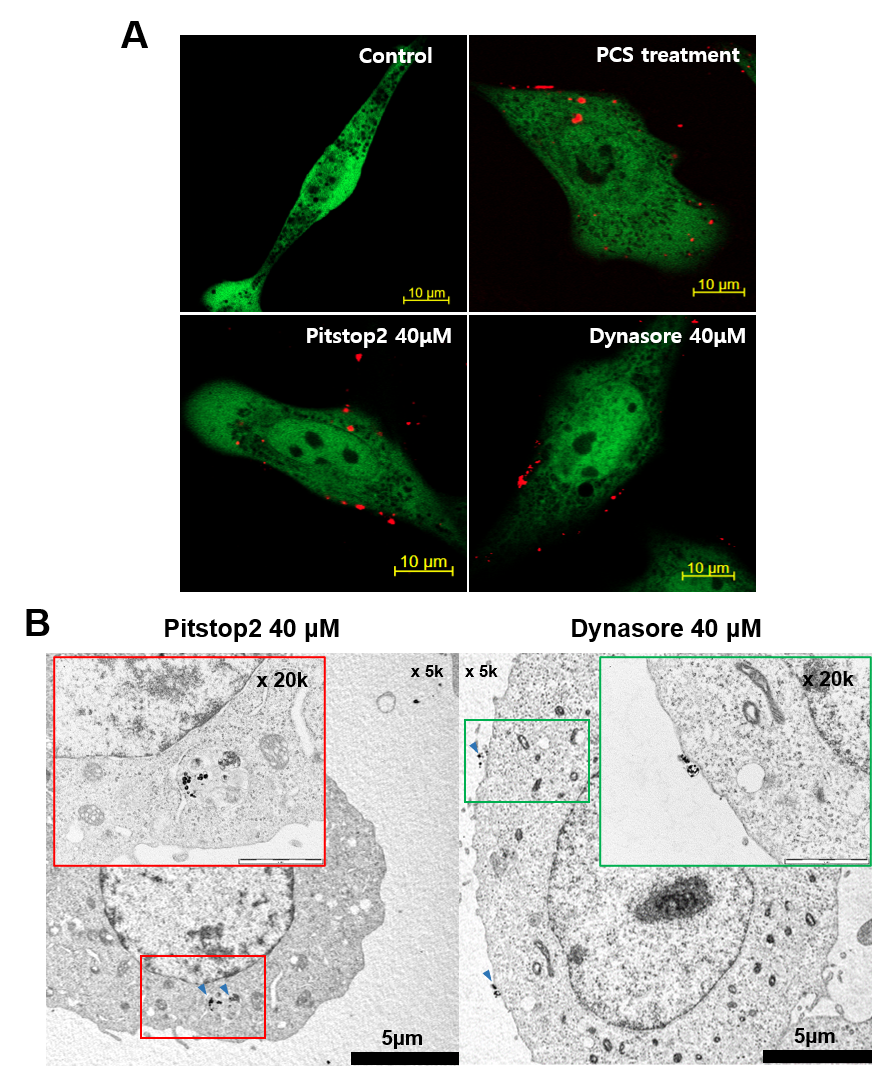
**

**Figure S8** Observation of PCS-NPs internalization after treatment of endocytic inhibitors. (A) Confocal image analysis of MSCs with inhibitors after 5 μg/mL PCS-NP treatment. (B) Intracellular observation of MSCs and PCS-NPs by TEM after treatment with the two internalization inhibitors.

**
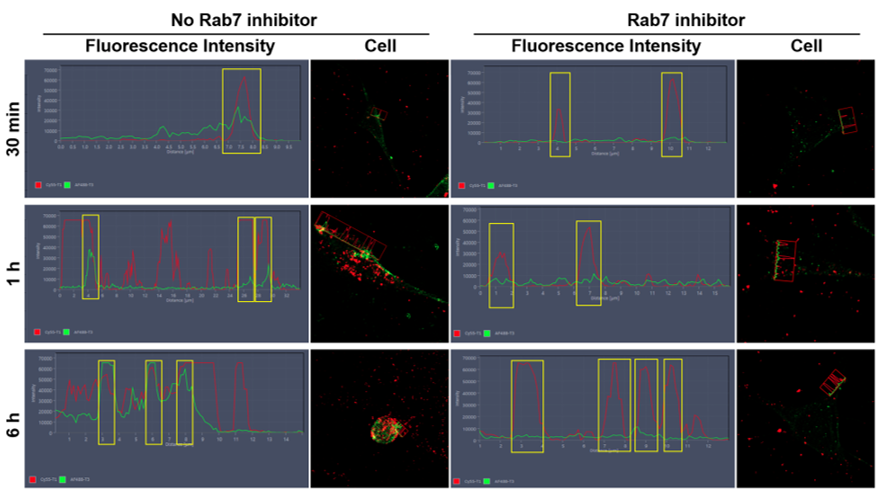
**

**Figure S9** Using Rab7 inhibitor, we confirmed the merge efficiency of intracellular nanoparticles with organelles. The fluorescence image analysis showed that the merge efficiency decreased between Rab7 and NPs in the inhibited cells, after exposure of 30 μM Rab7 inhibitor for 1 h. We determined the merge efficiency of cellular organelles with nanoparticles using confocal microscopy. Intensity was confirmed by cutting the fluorescent image horizontally. The nanoparticles (red) were increased, but organelles (green) did not express Rab7; therefore, nanoparticles did not enter the organelles after treatment with Rab7 inhibitor. The distances were plotted on the chart and averaged. The merge efficiency was analyzed by comparing the fluorescence intensity vacuoles of Rab7 expression (green) and nanoparticles (red) at the overlapping part of organelles and particles. When observed for 30 min, 1 h, and 6 h, it was clear that inhibition of Rab7 prevented nanoparticle transport to late endosomes and autolysosomes.

**
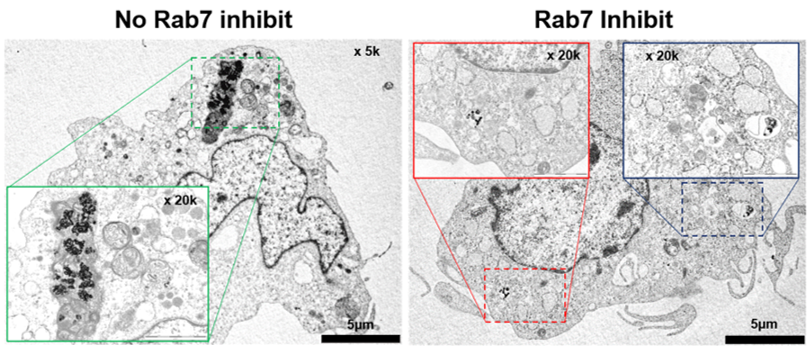
**

**Figure S10** Analysis of transportation pathway of PCS-NPs by Rab7 inhibitors by TEM. If the inhibitor is not reacted, NP enters the autolysosome-like organelle while the inhibitor does not proceed in the early stage.

**
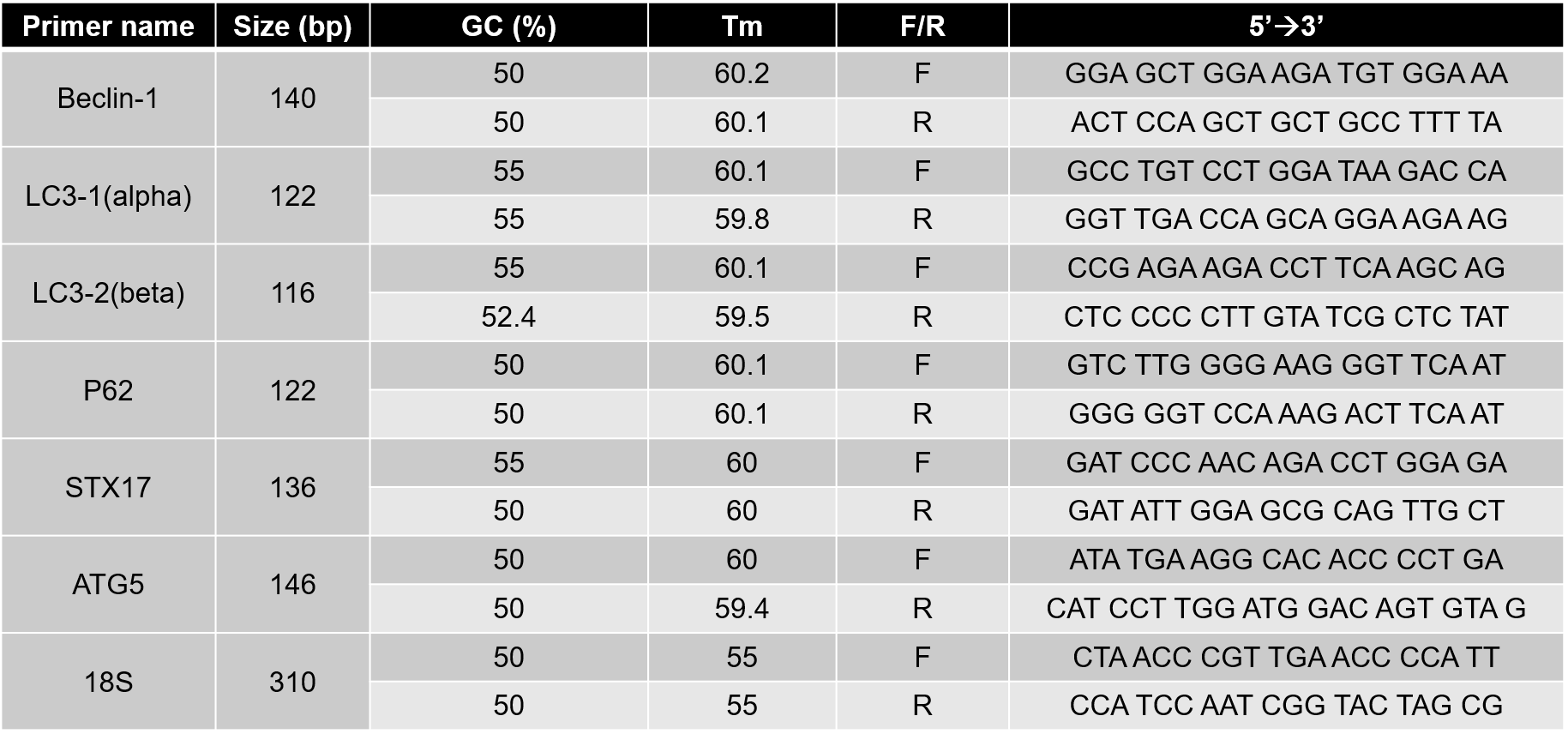
**

**Figure S11** List of primers related to autophagy. NCBI database was used to design the primers, and beclin-1, LC3-α, LC3-β, p62, STX17, ATG5, and 18S were chosen from relevant references.

**
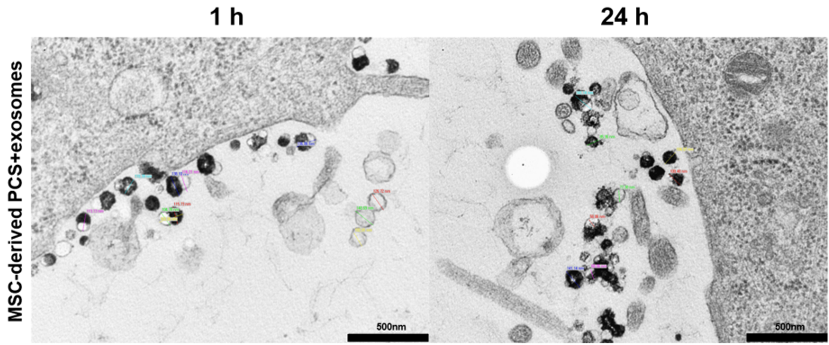
**

**Figure S12** Diameter of MSC-derived “PCS + exosomes” after magnetofection. The particles size at 1 h were 113.13, 104.78, 115.73, 138.1, 116.21, 110.39, 136.86, 126.72, 102.01 nm, and the average was 118.21 ± 13.02 nm. The particle sizes at 24 h were 77.38, 58.06, 141.14, 68.06, 100.48, 104.87, 85.36, 141.14 nm, and the average was 97.06 ± 31.27 nm.

**References**

[1] F. Danhier, E. Ansorena, J.M. Silva, R. Coco, A. Le Breton, V. Preat, PLGA-based nanoparticles: an overview of biomedical applications, J. Control. Release. 161 (2012) 505-522. <https://doi.org/10.1016/j.jconrel.2012.01.043>.

[2] A. E. Bashar, A.Metcalfe, A.Yanai, C. Laver, U.O. Hafeli, C.Y. Gregory-Evans, et al., Influence of iron oxide nanoparticles on innate and genetically modified secretion profiles of mesenchymal stem cells, IEEE Trans. Magn. 49(1) (2012) 389-393.

<https://doi.org/10.1109/TMAG.2012.2225829>

[3] A. Aires, D. Cabrera, L.C. Alonso‐Pardo, A.L. Cortajarena, F.J. Teran, Elucidation of the physicochemical properties ruling the colloidal stability of iron oxide nanoparticles under physiological conditions, Chem. Nano. Mat. 3(3) (2017) 183-189.

<https://doi.org/10.1002/cnma.201600333>

[4] X. Jiang, A. Musyanovych, C. Röcker, K. Landfester, V. Mailänder, G.U. Nienhaus, Specific effects of surface carboxyl groups on anionic polystyrene particles in their interactions with mesenchymal stem cells, Nanoscale, 3(5) (2011) 2028-2035.

<https://doi.org/10.1039/c0nr00944j>

[5] J. Gu, H. Xu, Y. Han, W. Dai, W. Hao, C. Wang, et al., The internalization pathway, metabolic fate and biological effect of superparamagnetic iron oxide nanoparticles in the macrophage-like RAW264. 7 cell, Science China Life Sciences, 54(9) (2011) 793-805.

<https://doi.org/10.1007/s11427-011-4215-5>

[6] V. Diana, P. Bossolasco, D. Moscatelli, V. Silani, L. Cova, Dose dependent side effect of superparamagnetic iron oxide nanoparticle labeling on cell motility in two fetal stem cell populations, PLoS One. 8(11) (2013) e78435.

<https://doi.org/10.1371/journal.pone.0078435>

[7] G.T. Noutsios, N. Thorenoor, X. Zhang, D.S. Phelps, T.M. Umstead, F. Durrani, J. Floros, SP-A2 contributes to miRNA-mediated sex differences in response to oxidative stress: pro-inflammatory, anti-apoptotic, and anti-oxidant pathways are involved, J. Biol. Sex Differ. 8(1) (2017) 37. <https://doi.org/10.1186/s13293-017-0158-2>

[8] A. Seiler, M. Schneider, H. Förster, S. Roth, E.K. Wirth, C. Culmsee, et al., Glutathione peroxidase 4 senses and translates oxidative stress into 12/15-lipoxygenase dependent-and AIF-mediated cell death, Cell Metab. 8(3) (2008) 237-248.

<https://doi.org/10.1016/j.cmet.2008.07.005>

[9] J.C. Hay, J. Klumperman, V. Oorschot, M. Steegmaier, C.S. Kuo, R.H. Scheller, Localization, dynamics, and protein interactions reveal distinct roles for ER and Golgi SNAREs, J. Cell Biol. 141(7) (1998) 1489-1502.

<https://doi.org/10.1016/j.cmet.2008.07.00510.1083/jcb.141.7.1489>

[10] L.Gerez, K. Mohrmann, M. van Raak, M. Jongeneelen, , X.Z. Zhou, K.P. Lu, P. van der Sluijs, Accumulation of rab4GTP in the cytoplasm and association with the peptidyl-prolyl isomerase pin1 during mitosis, Mol. Biol. Cell. 11(7) (2000) 2201-2211.

<https://doi.org/10.1091/mbc.11.7.2201>

[11] B. Du, W.P. Cawthorn, A. Su, C.R. Doucette, Y. Yao, N. Hemati, et al. The transcription factor paired-related homeobox 1 (Prrx1) inhibits adipogenesis by activating transforming growth factor-β (TGFβ) signaling, J. Biol. Chem. 288(5) (2013) 3036-3047. <https://doi.org/10.1074/jbc.M112.440370>

[12] S. Tamura, N. Matsumoto, R. Takeba, Y. Fujiki, AAA peroxins and their recruiter Pex26p modulate the interactions of peroxins involved in peroxisomal protein import, J. Biol. Chem. 289(35) (2014) 24336-24346. <https://doi.org/10.1074/jbc.m114.588038>

[13] S. Pabst, M. Margittai, D. Vainius, R. Langen, R. Jahn, D. Fasshauer, Rapid and selective binding to the synaptic SNARE complex suggests a modulatory role of complexins in neuroexocytosis, J. Biol. Chem. 277(10) (2002) 7838-7848.

<https://doi.org/10.1074/jbc.m109507200>

[14] D. Sica, P. Rayman, J. Stanley, J.H. Finke, E. Klein, E. Klein, et al., Interleukin 7 enhances the proliferation and effector function of tumor‐infiltrating lymphocytes from renal‐cell carcinoma, Int. J. Cancer. 53(6) (1993) 941-947.

<https://doi.org/10.1002/ijc.2910530613>

[15] C.M. Hales, R. Griner, K.C. Hobdy-Henderson, M.C. Dorn, D. Hardy, R. Kumar, et al., Identification and characterization of a family of Rab11-interacting proteins, J. Biol. Chem. 276(42) (2001) 39067-39075. <https://doi.org/10.1074/jbc.M104831200>

[16] A.A. Peden, E. Schonteich, J. Chun, J.R. Junutula, R.H. Scheller, R. Prekeris, The RCP–Rab11 complex regulates endocytic protein sorting, Mol. Biol. Cell. 15(8) (2004) 3530-3541. <https://doi.org/10.1242/jcs.032441>

[17] B.P. Zambrowicz, A. Abuin, R. Ramirez-Solis, L.J. Richter, J. Piggott, H. BeltrandelRio, et al. Wnk1 kinase deficiency lowers blood pressure in mice: a gene-trap screen to identify potential targets for therapeutic intervention, Proc. Natl. Acad. Sci. 100(24) (2003) 14109-14114. <https://doi.org/10.1073/pnas.2336103100>

[18] R. Greenlees, M. Mihelec, S. Yousoof, D. Speidel, S.K. Wu, , S. Rinkwitz, et al., Mutations in SIPA1L3 cause eye defects through disruption of cell polarity and cytoskeleton organization, Hum. Mol. Genet. 24(20) (2015) 5789-5804.

<https://doi.org/10.1093/hmg/ddv298>

[19] R.L.P. Santos-Cortez, K.Lee, A.P. Giese, M. Ansar, M. Amin-Ud-Din, K. Rehn, et al., Adenylate cyclase 1 (ADCY1) mutations cause recessive hearing impairment in humans and defects in hair cell function and hearing in zebrafish. Hum. Mol. Genet. 23(12) (2014) 3289-3298. <https://doi.org/10.1093/hmg/ddu042>

[20] B. Zhao, L. Chang, H. Fu, G.Sun, W. Yang, The role of autoimmune regulator (AIRE) in peripheral tolerance, J. Immunol. Res. 2018 (2018) e3930750.

<https://doi.org/10.1155/2018/3930750>

[21] E. Wyatt, R. Wu, W. Rabeh, H.W. Park, M. Ghanefar, H. Ardehali, Regulation and cytoprotective role of hexokinase III, PloS one 5(11) (2010) e13823.

<https://doi.org/10.1371/journal.pone.0013823>

[22] A. Sané, L. Ahmarani, E. Delvin, N. Auclair, S. Spahis, E. Levy, SAR1B GTPase is necessary to protect intestinal cells from disorders of lipid homeostasis, oxidative stress, and inflammation, J. Lipid Res. 60(10) (2019) 1755-1764. <https://www.jlr.org/content/60/10/1755>

[23] S.Y. Kim, S.M. Park, S.T. Lee, Apolipoprotein C-II is a novel substrate for matrix metalloproteinases, Biochem. Biophys. Res. Commun. 339(1) (2006) 47-54. <https://doi.org/10.1016/j.bbrc.2005.10.182>
